# Spatial Aggregations of the Grey Field Slug *Deroceras reticulatum* Are Unstable Under Abnormally High Soil Moisture Conditions

**DOI:** 10.3390/insects15100819

**Published:** 2024-10-19

**Authors:** Claire S. V. Price, W. Edwin Harris, Emily Forbes, Keith F. A. Walters

**Affiliations:** 1 Agriculture and Environment Department, Harper Adams University, Newport, Shropshire TF10 8NB, UK; cprice@harper-adams.ac.uk (C.S.V.P.); emily_forbes@hotmail.co.uk (E.F.); 2 Centre for Agriculture Data Science, Harper Adams University, Newport, Shropshire TF10 8NB, UK; eharris@harper-adams.ac.uk; 3 Joint Nature Conservation Committee (JNCC), Quay House, 2 East Station Road, Fletton Quays, Peterborough PE2 8YY, UK; 4 Department of Life Sciences, Imperial College London, Silwood Park Campus, Ascot, Berks SL5 7PY, UK

**Keywords:** precision agriculture, slug control, arable crops, climate change, waterlogging, integrated pest management

## Abstract

*Deroceras reticulatum* in arable fields display spatio-temporally stable slug patches that have been well documented under typical soil moisture conditions. The effect of abnormally high soil moisture on slug patch stability, however, is unknown. In this study, stepped gradient choice tests comparing soil moisture levels of 50–125% soil capacity showed slug preferences for levels in a range near to 125%. Activity became erratic, however, when given a choice of high moisture levels (125–370%), potentially because slugs searched for preferred conditions. Slug spatial aggregation was investigated in 21 commercial fields in 2023/24, a season of extreme rainfall, and then compared to years exhibiting typical rainfall (2015–2018). Slug patches occurred in 27.2% of assessment visits to fields during 2023/24 compared to 96.4% in typical years, suggesting weather conditions leading to abnormally high soil moisture are significantly associated with the breakdown of slug spatial aggregation behaviour. Random forest models identified the weather predictors (precipitation, relative humidity, temperature) with the highest impact on slug distribution and relative abundance, with the assessment date and region also related to relative abundance. However, a complex of environmental parameters affects soil moisture content, and no statistically significant effects of individual weather predictors emerged. The results are discussed in relation to slug behaviour in the context of their impact on targeted slug treatments.

## Simple Summary

The grey field slug is a pest of arable crops that is known for its heterogenous (patchy) distribution. In this study, the impact of abnormally high soil moisture on slug patch stability was investigated under laboratory conditions (using experimental soil moisture gradients) and under field conditions. In the laboratory, slugs preferred soil moistures near to 125% field capacity, but erratic movement patterns emerged when subjected to higher soil moisture. Under waterlogged field conditions, aggregated slug patches were found in only 27.2% of assessments (cf. 96.4% in earlier work under more typical field soil moisture conditions). The impact of various weather parameters on slug spatial aggregation was evaluated, with precipitation, relative humidity and temperature having the highest impact. In addition, the assessment date and region were also related to slug relative abundance. However, the complexity of environmental parameters affecting soil moisture content resulted in no individual weather factor emerging as a significant predictor of slug distribution. The results are discussed in relation to slug biology and behaviour and conclusions drawn on their impact on targeted slug control treatments. It was concluded that the spatial slug aggregations that occur under typical soil moisture conditions revert to a random distribution under high soil moisture conditions.

## 1. Introduction

The grey field slug *Deroceras reticulatum* (Müller, 1774; Agriolimacidae) is one of the most damaging pest species in arable crops in the UK [1] and other parts of the world [2,3,4]. Subject to favourable weather conditions, it can reproduce throughout the year, with two peaks of egg laying in autumn and spring, the former coinciding with the establishment phase of crops such as winter wheat and oilseed rape [5,6,7]. The slugs feed on seeds or the emerging plants, causing significant damage including crop failure, and without effective control it has been estimated that slug activity can result in an annual loss of GBP 43.5 million in British oilseed rape and wheat crops alone, rising to GBP 100 m in extreme years [8]. Control of the pest in the UK usually relies on conventional ferric phosphate pellets; for example, in 2022, molluscicides were used on 41% of the area of oilseed rape grown in the UK, with an average of two applications (214,778 ha), and 16% of the area of wheat grown in the UK (401,833 ha) [9].

The implementation of conservation agriculture practices such as minimum tillage, direct drilling, and cover crops have been linked to an increase in slug numbers in arable crops [10]. However, the removal from the market of methiocarb in 2015 and metaldehyde in 2022, due to perceived risks of environmental damage, resulted in there being only one conventional active ingredient (ferric phosphate) available for slug control in the UK, raising concerns relating to overreliance on a single active substance. [9,11,12,13,14,15]. A biological control agent is also available for use in the UK, the nematode *Phasmarhabditis hermaphrodita*, but this option is currently not cost-effective in arable crops and is aimed at high-value horticultural crops [11,12,13,16]. Reliance on a single control agent for the management of a major pest is associated with a range of risks, particularly where a large proportion of the crop is treated, and in the absence of available alternatives, other approaches are needed that can reduce product use [17].

Recent work has focused on the potential to reduce the quantity of molluscicidal products applied to arable fields while maintaining commercially viable slug control by exploiting the known heterogenous, spatially patchy distribution of the pest and its associated foraging [14,15,18,19,20]. It has been demonstrated that slug patches are characteristic of UK arable fields, that they are spatio-temporally stable throughout the slug damage windows of major crops, and they arise from behavioural responses resulting in stable slug patch formation [14,20]. In silico research has demonstrated that these behavioural characteristics alone result in steady-state aggregation indices that are reflected in those found in commercial arable fields [20,21,22]. This distribution is reinforced in part by a tendency to lay egg masses in batches, leading to juvenile aggregations [15,18], and it facilitates the tendency to group together to reduce water loss under adverse conditions such as dry weather [23]. Comparing the areas of commercial fields with and without slug patches suggests that by applying control measures to only the slug patches (patch treatment) would yield a saving in the amount of product applied of up to 40–50%, offering significant environmental advantages and lower input costs [24].

A limitation of past studies of slug patch formation and stability is that field work has been conducted under a limited range of weather conditions, and little is known about the effect of exceptionally high rainfall, leading to abnormally high soil moisture content and waterlogging, on slug spatial aggregation behaviour. This focus is reflected in work on other aspects of slug biology [2,15,25,26]. Previous laboratory studies have investigated the effect of high soil moisture (including waterlogging) on *D. reticulatum* activity, which is known to vary the number of eggs laid depending on moisture levels [5]. Further, Carrick [25] showed that the eggs of *Deroceras* (previously *Agriolimax) agrestis* laid in 100% water-saturated soil did not hatch, with the development of the majority of embryos terminating at early stages. The author theorised that between 40 and 80% saturation was ideal for the laying and successful development of eggs. 

The specific behaviour leading to slug patch formation and stability has been established in previous work and is triggered by encounters with conspecifics [22,23]. These conspecific responses change key components of slug movement (mean speed, turning angles, bias in turning direction, movement and resting times), collectively causing *D. reticulatum* to forage within a limited area and resulting in the formation of aggregations of the sizes found in arable crops. If submerged in water for a sufficient time, however, slugs have been shown to drown [2], and it is hypothesised that when soil is waterlogged, they may revert to moving randomly until they locate drier soils, thus increasing their probability of survival and successful reproduction but resulting in patch breakdown. If this is correct, as soils dry, then slug patches may ultimately start reforming as conspecific responses are restored.

As climate change amplifies the severity and frequency of extreme weather events (e.g., excessive rainfall and flooding [27,28]), the impact of waterlogging on the ability of *D. reticulatum* to form high-density patches in arable fields will become an important factor that future patch treatment approaches will need to incorporate. Between October 2023 and March 2024, the UK experienced unusually high rainfall, 60% above the last 10-year average, with a significant impact on farming such as flooding, soil erosion and damage to crops [29] and with the potential effect of de-stabilising *D. reticulatum* high-density patches.

This study investigates three hypotheses: (i) under laboratory conditions, slugs will show a preference for a soil moisture that is neither too dry or too wet; (ii) slug patch formation and spatial stability are negatively affected by waterlogging in commercial fields, leading to a random distribution of the slug population; and (iii) weather factors that will influence soil moisture content in the field (temperature, relative humidity and precipitation) will be potential predictors of slug relative abundance and distribution.

## 2. Materials and Methods

### 2.1. Laboratory Soil Moisture Experiment

#### 2.1.1. Experimental Design

Adult *D. reticulatum* (3–4.5 cm long) were collected from two field sites located in Shropshire (52°46′01.26″ N 002°34′50.14″ W) and Lancashire (53°30′22.66″ N 002°42′25.54″ W) and returned to a constant environment (CE) room in the laboratory (Sanyo SGC097.CFX.F Fitotron Temperature/Humidity Test Chamber, Weiss Technik UK Ltd., Loughborough, UK) for a 48 h acclimatisation period. During this period, the slugs were maintained under standard conditions of 14 h:10 h light/dark, 15 °C (photophase)/10 °C (scotophase), and 60% relative humidity. 

Individual slugs were maintained in a 250 mL circular plastic container (11.5 cm diameter; 4.2 cm high) with eight 1 mm diameter holes drilled through the lid for ventilation. The base of each container was covered with a 4 cm^2^ disc of damp paper towel (2-ply blue centrefeed roll, Cater4you, High Wycombe, UK), moistened with 1 mL of distilled water, which was replaced daily. Slugs were fed ad libitum on 1 cm thick slices of carrot that were also replaced daily. 

Slug preferences for different soil moisture levels were investigated by establishing two soil moisture gradients consisting of four discrete levels, attributed to either a low- or high-moisture treatment (Table 1). Each gradient consisted of four compartments (15 cm × 22 cm × 3.5 cm) made from foil trays (D-272-33, Cater4You, UK), connected by making 2.5 cm cuts in the corners of the long (22 cm) edges and folding them to create a smooth joint between adjacent compartments (see Figure S1 Supplementary Material). A 2 cm wide band of a Vaseline and salt mixture (ratio 4:3) was applied to the edge of the gradient (but not the connecting platform) of each compartment to contain the slugs within the experimental arena. 

The soil substrate (clayey loam) for the compartments was collected from the Harper Adams University campus (52°46′01.26″ N 002°34′50.14″ W). The substrate was oven-dried for 72 h at 35 °C (Genlab Multi-Purpose Oven (LCO/42H/DIG, Genlab, UK)) and sieved using a 2 mm wide mesh. Each compartment was weighed before a 1 cm deep layer of the soil was added, with its surface level with the joint between the compartments. The compartments were then reweighed to establish the exact weight of the soil contained. Distilled water was added to individual compartments to elevate the moisture content and create the gradients described in Table 1. The amount of water added was calculated using the method of Black (1965) [30]. A total weight (including foil tray, soil, Vaseline/salt and water) was recorded for each compartment, to allow for any water lost through evaporation to be replaced at daily intervals. Food consisted of one carrot slice (2 cm diameter, 0.5 cm thickness) placed in the centre of each compartment, which ensured excess food was available throughout the experiment. The two treatments were run simultaneously, with an individual slug released in a randomly assigned (using a random number generator) compartment. The low-moisture treatment was replicated twenty times, and the high-moisture treatment was replicated eight times only, due to slug availability.

During all experimental replicates, the stepped gradients were maintained under the standard conditions defined above. Each replicate commenced one hour after the start of scotophase as slugs are active at night [25], and the position of each slug within each compartment was recorded three times per day at 1, 5 and 9 hrs after the start of scotophase for five days.

#### 2.1.2. Statistical Analysis—Soil Moisture Gradient Experiment

Data analysis was conducted using R (version 4.4.0) [31]. The proportion of slugs distributed among the different soil moisture gradients was analysed using a Kruskall–Wallis rank sum test, and group comparisons were made using Dunn’s post hoc test. 

### 2.2. Distribution of Slugs in Commercial Arable Fields

#### 2.2.1. Field Sites

The degree of spatial aggregation of the slugs was investigated in 21 commercial field crops grown in the major arable regions of England (Table 2). Slug assessments were conducted during a minimum of three and a maximum of seven visits to the experiment, commencing one to two weeks after crop emergence and thereafter at approximately 14-day intervals. Assessments were made during autumn and winter 2023/2024 at all except three sites (Cambridgeshire 1, Hampshire and Oxfordshire 2), where they were made in spring 2024 after the loss of the autumn-sown crop due to waterlogging necessitated re-drilling [29]. Waterlogged or near-waterlogged fields following heavy rainfall in the autumn also delayed access, preventing strict adherence to the planned schedule of assessments. 

#### 2.2.2. Experimental Design

The experimental design used to assess slug activity and spatial distribution on the soil surface followed a method developed and successfully tested in previous research [32,33]. A 1 ha plot containing a 100 m × 100 m grid of surface refuge slug traps (10 m between nearest neighbours) was established in each field, and the location of the corners of the grid noted using a global positioning system (GPS). The traps consisted of upturned terracotta-coloured plastic plant pot saucers (18 cm diameter, LBS Horticulture Supplies, Lancashire, UK) and were not baited to avoid slug feeding attraction. Each trapping grid was sited at a minimum of 20 m from the field boundary to reduce the potential for the perimeter-to-core slug density gradients to differentially affect the accuracy of patch identification across the grid [34]. Each trap was allotted a unique code indicating its position in the trapping grid, to record slug aggregations spatially. At each assessment (commencing at approximately 09:00), the number of *D. reticulatum* found under each trap (i.e., on the soil and the surface of the trap) was recorded. The trap was then immediately replaced, with any captured slugs released beneath it. The slug count was recorded for each trap location and sampling occasion using a custom phone application. 

#### 2.2.3. Statistical Analysis—Field Experiment

Data analysis was conducted using R (version 4.4.0) [31]. Slug distributions within the trapping grid were visualised for each sampling visit to the crops studied using grid maps of counts, with the relative abundance of slugs (log-transformed) in areas between traps estimated by polynomial interpolation. To describe the slug distribution, the SADIE (Spatial Analysis by Distances Indices [35]) index of aggregation I_a_ was computed and evaluated as a measure of model performance. 

Following tests for the normality and heterogeneity of the data, the effect of the region and date on slug relative abundance and slug distribution were analysed with a general linear model (GLM). The effect of the current crop was not investigated as all fields were growing winter wheat except for Cambridgeshire 1, Hampshire and West Sussex, resulting in an unbalanced variable distribution that would lead to biased results. The effect of the previous crop was not investigated as several crops such as winter beans, oats, peas, birdsfoot trefoil and legume fallow were each grown in only one of the 21 fields.

Weather data, including the mean and sum of temperature, mean relative humidity and sum of precipitation each for one, two, three and four weeks before each assessment date, were derived a posteriori using hourly spatial temperature (2 m above ground), relative humidity (2 m above ground) and precipitation (mm/day) data from {nasapower} in R with a spatial resolution of 0.5 × 0.625 degrees latitude and longitude [36], using what3words (https://what3words.com) coordinates of each field, provided by the growers. To preserve the anonymity of the host growers, geolocation coordinates are not reported for fields in the present study. The data from nasapower were not compared with measurements on the ground. Weather data were combined with coordinates, soil type and date and analysed using random forest models, specifically as a performance indicator to guide the selection of variables to include in the next stage of the analysis [37]. The top predictors for each weather variable were then included in a linear mixed-effects model (with individual fields treated as a random effect) for slug relative abundance and in a regression analysis for slug distribution (clumped or random). Weather parameter values for each occasion were averaged based on distribution (clumped or random) and compared using a paired *t*-test. 

## 3. Results

### 3.1. Laboratory Soil Moisture Experiment

**Figure 1:**
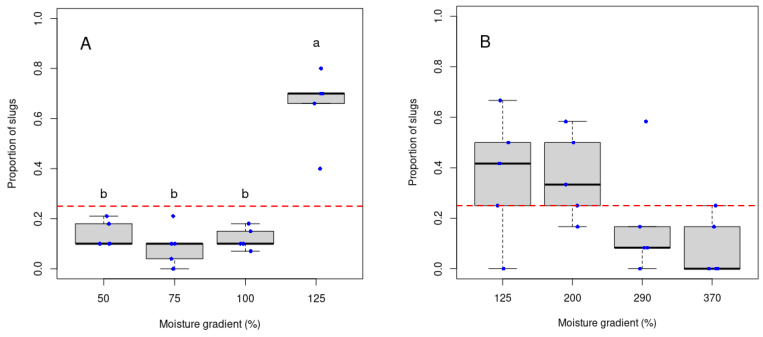
Proportion of *D.* reticulatum found on soil with different moisture gradient levels: low-moisture treatment between 50 and 125% field capacity ((**A**), *n* = 20), and high-moisture treatment between 125 and 370% field capacity ((**B**), *n* = 8) at the end of the five-day experiment. The different letters represent significant differences between groups identified by Dunn’s post hoc test. The red line represents the overall mean of the data. The blue dots represent data points.

At the end of the five-day experiment, the adult *D. reticulatum* showed a significant preference for soil at 125% field capacity in the low-moisture treatment (Figure 1A; Kruskal–Wallis chi-squared = 12.0279, df = 3, *p* < 0.05), with a lower proportion of slugs present when the soil moisture was lower (50–100%). In the high-moisture treatment, however, no preference was shown between the soil moisture levels (Figure 1B; Kruskal–Wallis chi-squared = 6.3694, df = 3, *p*-value = 0.09).

**Figure 2:**
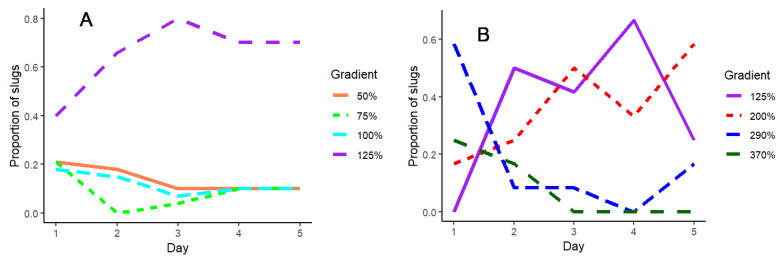
The proportion of *D. reticulatum* recorded on each day of a five-day experimental period, in different compartments of the moisture gradients containing soils with a different moisture content. The gradients compared soil moistures of (**A**) 50 to 125% field capacity and (**B**) 125 to 370% field capacity.

Slug movement between gradients over the five-day period is illustrated in Figure 2A,B and shows that while slug movement stabilised in all gradients from day three onwards when lower soil moisture levels were tested (Figure 2A), the movement was more erratic in the high moisture gradient (particularly in a range around 125% and 200% field capacity) across the five days (Figure 2B).

### 3.2. Distribution of Slugs in Commercial Arable Fields

Slug distribution was found to be significantly clumped on 22 sampling occasions, with a random distribution on 81 sampling occasions. On two dates, the distribution was not evaluated as no slugs were present. Slug distribution was random for all sampling occasions in 7 fields out of 21 (Cambridgeshire, Hampshire, Herefordshire, Hertfordshire 1, Lincolnshire 2, Rutland, Shropshire), and no field had a clumped distribution across all dates (see Figures S2–S22 in Supplementary Material).

No effect of date and region was found on slug distribution, but the number of slugs recorded in traps (relative abundance) was significantly influenced by regions (F-value = 4.33, df = 16, *p* < 0.05), with higher slug trap catches in Northamptonshire (t-value = 2.52, *p* < 0.05) and lower in West Sussex (t-value = −2.05, *p* < 0.05). Slug abundance was also influenced by assessment dates and decreased steadily with the passing of time (between October 2023 and June 2024; F-value = 12.9, df = 1, *p* < 0.05).

The random forest analysis for slug relative abundance explains 11.8% of the variation in the data. The predictors with the highest impact on slug abundance for each weather data category were the average relative humidity (RH) for two weeks before the slug assessment was made, the sum of temperatures (or degree days) for four weeks before the slug assessment and the sum of precipitation for one week before the slug assessment (italicised in Table 3), and individual fields are also a strong predictor of slug abundance. The linear mixed-effect model fixed effects results are presented in Table 4. As a random effect, individual fields accounted for 47% of variance.

The error rate of the random forest models for slug distribution amounted to 24.75%, and the error rate for the clumped class amounted to 85.71% while for the random class it was 8.75%. Thus, the model is biased towards predicting the random class due to low numbers of clumped instances. The predictors identified by the random forest models as having the highest impact on slug distribution were the sum of precipitation for four weeks before the slug assessments, mean relative humidity for two weeks before the slug assessments and the sum of the temperature for three weeks before the slug assessments (italicised in Table 3). The regression analysis results are presented in Table 5.

Slug distribution types (clumped or random) when compared with each other using averaged weather parameter values did not show significant differences (t = 1.69, df = 15, *p* = 0.11).

## 4. Discussion

Work on slug patches to facilitate precision pest management has focused on major commodities such as cereals and oilseed rape and shown the formation of slug aggregations to be typical in the UK, with similar slug distribution patterns also being recorded in a range of minor crops [20,32]. The effective targeting of these patches with pesticides relies on them being spatio-temporally stable throughout the slug damage window of the crop. This has been demonstrated empirically in arable fields, and the underpinning behavioural mechanisms resulting in both stability and the initial patch formation have also been established [14,20,22,23]. Density-dependent responses are stimulated when slugs detect conspecifics leading to a lower movement speed and time spent moving, more frequent turns, increased turning angles and a turning direction bias, collectively acting to maintain individuals within a localised area. 

Terrestrial slug activity and population distributions in commercial fields are also affected by soil moisture, however, and when individuals encounter sub-optimal conditions, they can move to areas with a more favourable moisture content to survive [26,39,40,41,42,43,44]. Individuals have also been shown to retreat underground (e.g., in response to dry soil conditions [2,15] and high temperatures [2,25]), as environmental variables are more stable below the soil surface [42]. Although it has been demonstrated that soil moisture is correlated with both slug numbers and level of activity and that it affects the locations in which higher-density slug patches form [25,26,42,43,44], field studies have not hitherto investigated the effect of extreme physical conditions such as very high soil moisture and waterlogging on the patch-forming behaviour of slugs, and thus their stability. Laboratory experiments in this study indicate that adult *D. reticulatum* display a preference for a soil water content near to 125% field capacity, when compared with lower and higher moisture levels. When only offered options of soil moisture content of 125% field capacity or higher (up to 370%), less consistent choices were made, suggesting the movement to locate optimal conditions was more dynamic and continued for longer. The responses to the lower soil moisture levels reflect those of other studies [2,15,34]. However, as slugs have been shown to drown if submerged for long periods in standing water [2], it is likely that under waterlogged conditions different responses to soil water content will be recorded, resulting in slugs moving to the soil surface and displaying more spatially dynamic movement as they disperse across the field to seek dryer areas. In addition, the observation that slugs can lay eggs throughout the year whenever conditions are favourable, but on water-saturated soil some species suffer adverse effects on subsequent development and hatching, reinforces this hypothesis [5,6,25].

The exceptionally high rainfall encountered in the UK during the autumn 2023 and winter 2024 provided an opportunity to investigate the effect of extreme weather conditions on patch formation and stability in the field. Previous work conducted during a similar period of the year (autumn and winter) in the same regions of the UK and using similar methodology, but under far less extreme weather conditions, recorded patchy slug distributions in 96.4% of the assessment visits [20], much higher than the 21% reported in the current study. The small proportion of assessment visits where a random distribution was recorded in the previous study were all associated with periods of low slug surface activity in the field, linked to high temperatures and low rainfall. The prevalence of random distributions in the current work could only be generated if the conspecific responses that result in the maintenance of slugs in higher-density patches were replaced by the resumption of the more linear tracks and longer time spent moving that are more usually described during periods when there are no encounters with conspecifics [22]. Following exposure to high soil water content in the laboratory experiment, slug movement was more dynamic and continued for longer, suggesting an avoidance response that was reflected in the field study and resulted in slugs moving around the field in search of favourable soil moisture levels. If large areas of a field have persistently waterlogged soil, the collective result of these behavioural changes would lead to a period of more random distribution of slugs. The steady decline in slug populations identified throughout the assessment period indicates a suppressed survival rate, which may drive such behavioural changes.

Our random forest results ranked weather parameters such as temperature, relative humidity and precipitation as explaining the highest amount of variation for slug relative abundance, though some of these were not statistically significant when subjected to further analysis. These will affect the level of soil moisture through the deposition or evaporation of water and potentially contribute to the triggering of the behavioural changes described above. The low variance explained by the models and the biased error rates, however, show that these factors alone do not adequately explain the variations in slug relative abundance and slug distributions, and consequently, more parameters would be needed to increase the predictive power of the model. The spatial resolution of the weather data could also have limited the accuracy of the results, but measurements taken in the fields were not available to compare them with.

The rate of drying of soil is influenced by a wide range of factors including (amongst others) the initial soil moisture, potential evaporation, vegetation cover, soil texture and organic matter content [45]. Soil moisture varies following rainfall events, and evaporation is affected by weather factors such as relative humidity and temperature. Thus, although contributing to changing slug distribution patterns, over a timescale concomitant with this study the individual impacts of various weather factors on the overall rate of change of slug distribution is likely to be low and difficult to quantify. Soil structure and organic matter also affect moisture retention capacity, and under less extreme conditions have been linked to the location of higher slug density patches in commercial fields [46]. Slugs are inefficient regulators of their body water content, resulting in a reliance on environmental moisture, and their movement is arrested by areas of the field with higher moisture retention capacities [34]. However, following periods of waterlogging, the soil structure may operate differently. Survival may be dependent on locating areas of the field that dry more quickly, as suggested by the laboratory experiment. 

In conclusion, this study indicated that under laboratory conditions slugs display a preference for a soil moisture of around 125% field capacity, with fewer individuals selecting soils with a lower or higher moisture content. Thus, the first hypothesis investigated was supported. Under the high soil moisture conditions encountered in commercial fields in 2023/24, slug patch formation was detected in fewer assessment visits when compared with historical data from years with less extreme conditions. It was concluded that patch formation and spatial stability were negatively affected by waterlogging in commercial fields leading to a random distribution of the slug population, supporting the second hypothesis. Predictors identified as having the highest impact on slug abundance included weather factors that influence soil moisture content in the field (temperature, relative humidity and precipitation). However, statistical significance was not recorded in most cases, due to the complex of other factors that also affect soil moisture. Thus, the third hypothesis was only partially supported. The low prevalence of stable slug patches in fields with abnormally high soil moisture will prevent the effective targeting of such patches to reduce molluscicide usage in commercial crops. However, the most commonly used control agents in Europe are slug pellets which are applied using a tractor-mounted spreader, and crop damage will deter access to the field under such conditions. Thus, the findings are unlikely to have a significant negative affect on the principle of improving sustainable pesticide use by patch treatment.

## Figures and Tables

**Table 1:** Levels of soil moisture (% field capacity) investigated in the gradients to establish substrate preferences of adult grey field slugs, *Deroceras reticulatum*.

Treatment Range	Compartment 1	Compartment 2	Compartment 3	Compartment 4
Low moisture	50%	75%	100%	125%
High moisture	125%	200%	290%	370%

**Table 2:** Field codes, the town nearest to the field site, the crop grown during the experiment, the previous crop in the rotation and the soil type for each field used in the experiment investigating the distribution of *D. reticulatum* in arable crops in 2023/2024.

County/Field Code	Nearest Town	Crop	Previous Crop	Soil Type
Bedfordshire	Riseley	Winter Wheat	Wheat	Heavy chalky boulder clay
Cambridgeshire 1	Sawtry	Spring Wheat	Oats	Heavy clay
Cambridgeshire 2	Cambourne	Winter Wheat	Winter Wheat	Hanslope series clay
Hampshire	Overton	Spring Barley	Winter Wheat	Silty loam
Herefordshire	Lea	Winter Wheat	*Data not available*	Clay loam
Hertfordshire 1	Ware	Winter Wheat	Oilseed Rape	Medium loam
Hertfordshire 2	Walkern	Winter Wheat	Oilseed Rape	Sandy silt loam
Kent	Lenham	Winter Wheat	Oilseed Rape	Silty clay loam
Leicestershire	Lutterworth	Winter Wheat	Oilseed Rape	Sandy loam
Lincolnshire 1	Wold Newton	Winter Wheat	Oilseed Rape	Medium clay loam
Lincolnshire 2	Louth	Winter Wheat	*Data not available*	Clay loam
North Yorkshire	Thirsk	Winter Wheat	Oilseed Rape	Medium loam
Northamptonshire	Cogenhoe	Winter Wheat	*Data not available*	Clay loam
Nottinghamshire	Farnsfield	Winter Wheat	Oilseed Rape	Clay loam
Oxfordshire 1	Faringdon	Winter Wheat	*Data not available*	Clay loam
Oxfordshire 2	Faringdon	Winter Wheat	Legume Fallow	Sandy loam brash
Rutland	Empingham	Winter Wheat	Peas/birdsfoot trefoil	Sandy clay loam
Shropshire	Much Wenlock	Winter Wheat	Oilseed Rape	Silty loam
Suffolk	Great Waldingfield	Winter Wheat	Dried Winter Bean	Clay loam/sandy silt loam
West Sussex	Petworth	Rye/Vetch	Wheat	Sandy loam
Yorkshire	Shipton	Winter Wheat	Oilseed Rape	Clay loam

**Table 3:** The increase in mean square error (%IncMSE) and the Increase in Node Purity values of weather variables from random forest models showing their relative impacts on the variation in slug relative abundance recorded in the surface refuge traps, and the subsequent slug distribution (prec = precipitation in mm/day; T = temperature in °C at 2 m above ground level; RH = percentage of relative humidity at 2 m aboveground). The increase in mean square error (%IncMSE) is based upon the mean decrease in accuracy in the predictions on the out-of-bag samples when a given variable is permuted [38]. Variables are ranked by values of %IncMSE. Italicised text represents the predictors with the highest impact that were selected for the mixed-effect model and regression analysis.

Slug Relative Abundance		Slug Distribution	
Variable	%IncMSE	Variable	%IncMSE
*RH_mean_2w*	*83.2*	*prec_sum_4w*	38.4
RH_mean_4w	72	*RH_mean_2w*	35.3
RH_mean_3w	56.2	*T_sum_3w*	30.6
*T_sum_4w*	*51.3*	T_mean_3w	29.7
T_mean_4w	50.4	T_mean_4w	27.4
*prec_sum_1w*	*48.6*	T_sum_4w	27.1
T_sum_3w	44.5	RH_mean_4w	25.4
T_mean_3w	44.3	T_sum_1w	23.9
T_mean_2w	39.9	RH_mean_3w	22.8
T_sum_2w	39.2	T_mean_1w	21.2
T_sum_1w	38.5	prec_sum_3w	14.8
T_mean_1w	36.7	prec_sum_1w	14.4
RH_mean_1w	26.7	T_mean_2w	10.5
prec_sum_4w	26.2	T_sum_2w	10.4
prec_sum_3w	18.5	RH_mean_1w	8.5
prec_sum_2w	5	prec_sum_2w	3.7

**Table 4:** Mixed-effect linear model variable results on slug relative abundance. RH_mean_2w = average relative humidity two weeks before slug assessments; T_sum_4w = sum of temperature four weeks before slug assessments; prec_sum_1w = sum of precipitation one week before slug assessments.

Term	Estimate	Standard Error	Statistic	*p*
(Intercept)	−1.360	5.330	−0.260	0.800
RH_mean_2w	0.058	0.053	1.100	0.280
T_sum_4w	0.001	0.002	0.250	0.800
prec_sum_1w	−0.003	0.008	−0.330	0.740

**Table 5:** Mixed-effect linear model variable results on slug distribution. prec_sum_4w = sum of precipitation four weeks before slug assessments; RH_mean_2w = mean relative humidity two weeks before slug assessments; T_sum_3w = sum of temperature three weeks before slug assessments.

Term	Estimate	Standard Error	Statistic	*p*
(Intercept)	3.530	13.503	0.261	0.794
prec_sum_4w	−0.006	0.009	−0.705	0.481
RH_mean_2w	−0.014	0.137	−0.103	0.918
T_sum_3w	−0.001	0.008	−0.123	0.902

## Data Availability

The datasets used and/or analysed during the current study are available from the corresponding author on reasonable request.

## Supplementary Materials

The following supporting information can be downloaded at https://www.mdpi.com/article/10.3390/insects15100819/s1, Figure S1: Design of the stepped moisture gradient apparatus used in laboratory studies of slug preferences for, and behavioural responses to, different soil moisture levels; Figures S2–S22: The distribution of *Deroceras reticulatum* within the trapping grid at the 21 field sites each with multiple assessment date.
