# Supplementary material for: Spatial Aggregations of the Grey Field Slug Deroceras reticulatum Are Unstable Under Abnormally High Soil Moisture Conditions
Source: Insects. 2024 Oct 19;15(10):819. doi: 10.3390/insects15100819 (PMC11508239; doi:10.3390/insects15100819)
Supplement: Supplementary file 1 [file insects-15-00819-s001.zip › insects-3233307-supplementary.pdf]

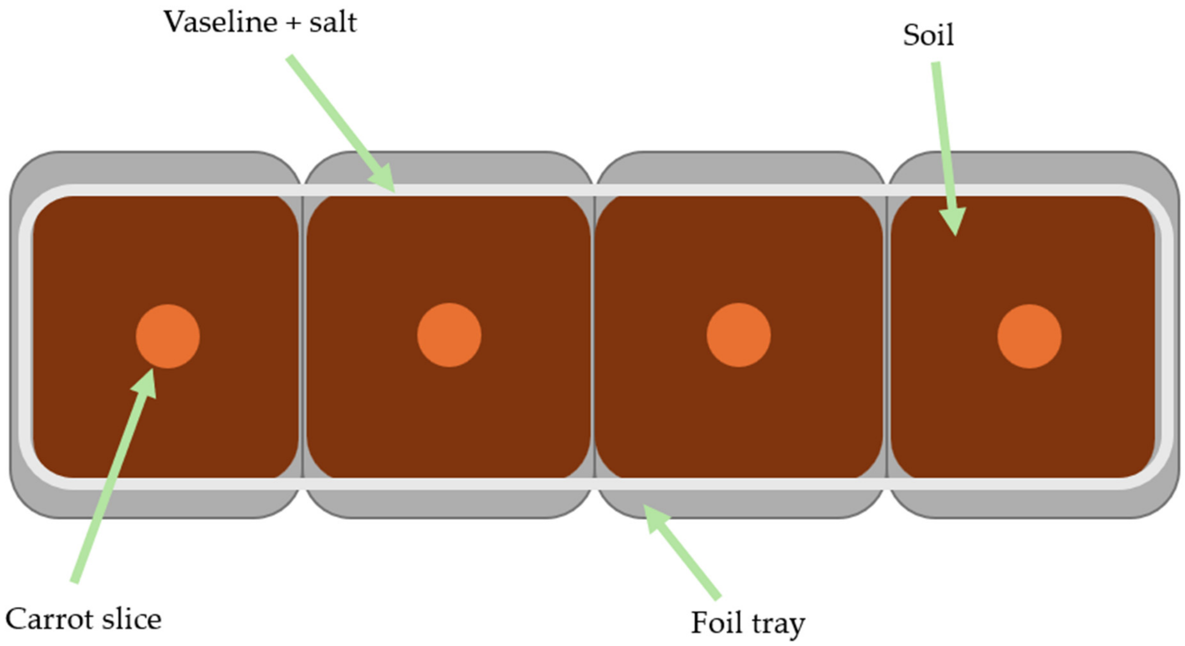

Figure S1. Design of the stepped moisture gradient apparatus used in laboratory studies of slug preferences for, and behavioural responses to, different soil moisture levels. Each of the four compartments consisted of a foil tray (15 cm x 22 cm x 3.5 cm), connected by making 2.5 cm cuts in the corners of the long (22 cm) edges and folding the resultant flaps to create a smooth joint between adjacent trays. A 2-cm-wide band of Vaseline and salt mixture (ratio 4:3) applied to the edge of the gradient (but not the connecting joint between each compartment) contained the slugs within the experimental arena. Each compartment contained a 1 cm deep layer of soil (with its surface level with the joint between the trays), each with sufficient distilled water added to maintain the soil moisture level required to establish a four-step moisture gradient.

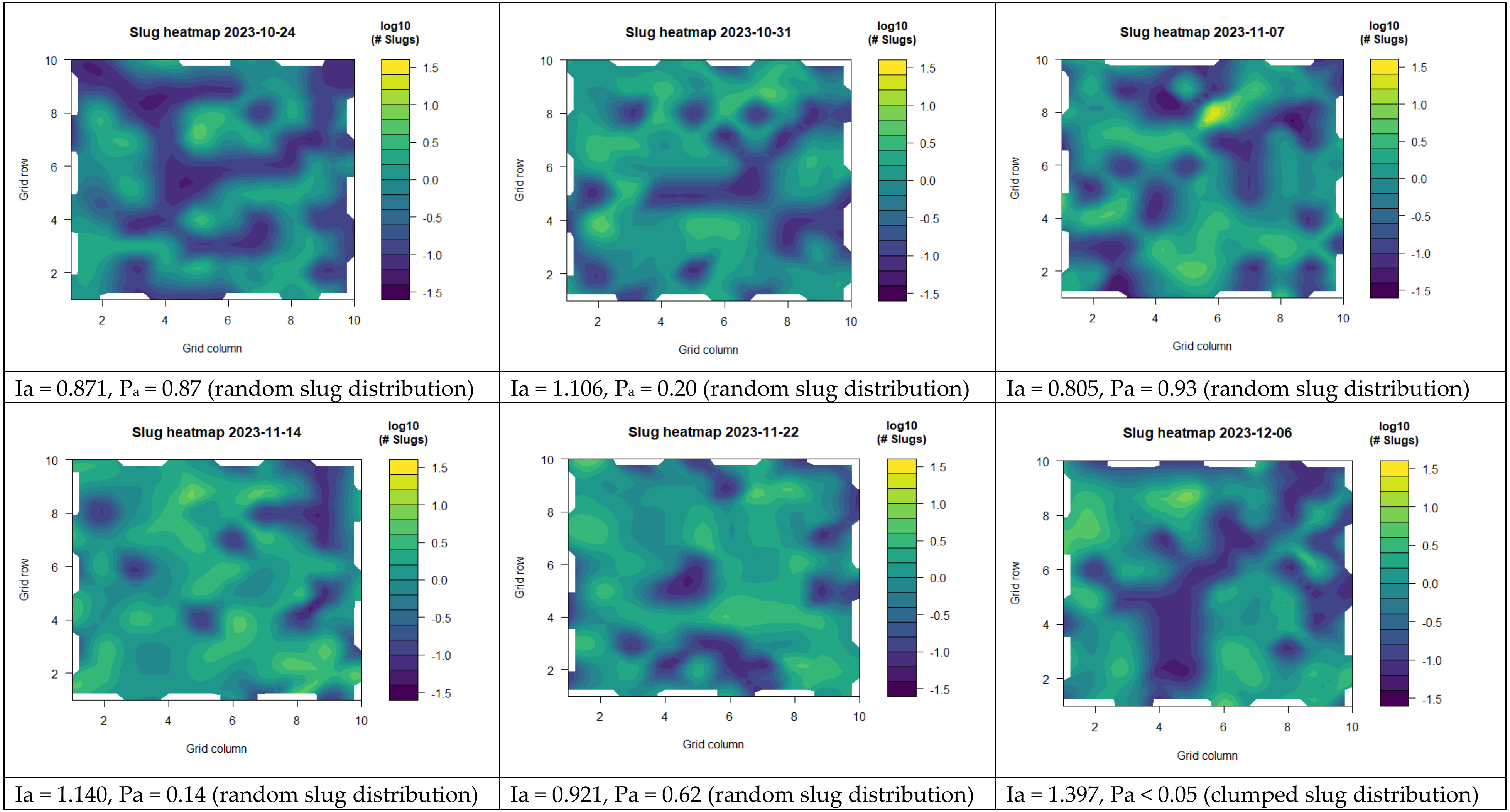

Figure S2. The distribution of *Deroceras reticulatum* within the trapping grid at the Bedfordshire field site for each assessment date (slug numbers log-transformed). Ia = SADIE (Spatial Analysis by Distances Indices) index of aggregation; Pa = probability level. Slug distribution based on values of Ia and Pa is indicated in brackets.

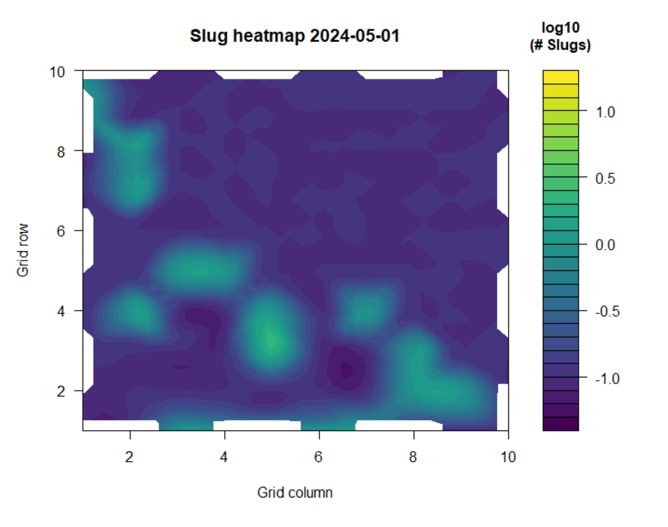

Ia = 1.513, Pa < 0.05 (clumped slug distribution)

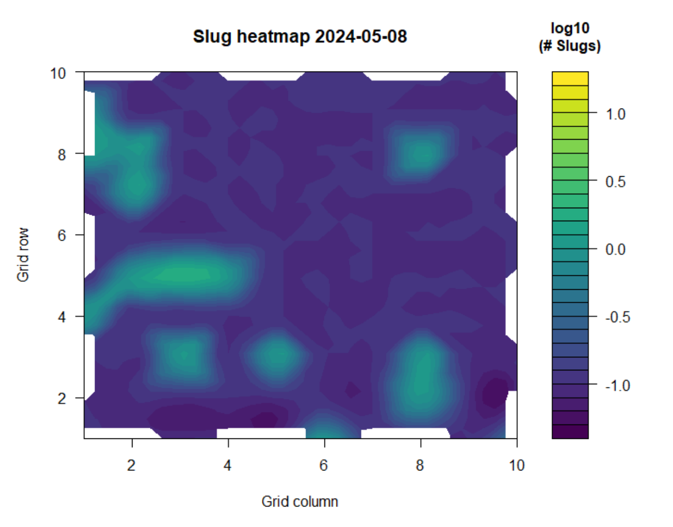

Ia = 1.198, Pa = 0.15 (random slug distribution)

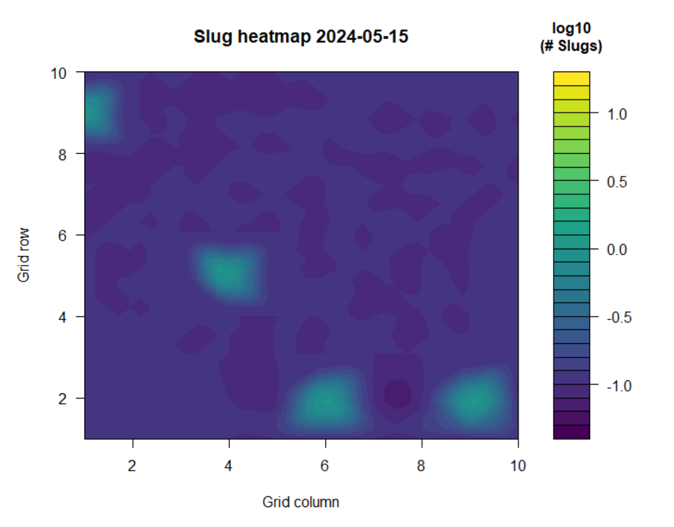

Ia = 0.950, Pa = 0.56 (random slug distribution)

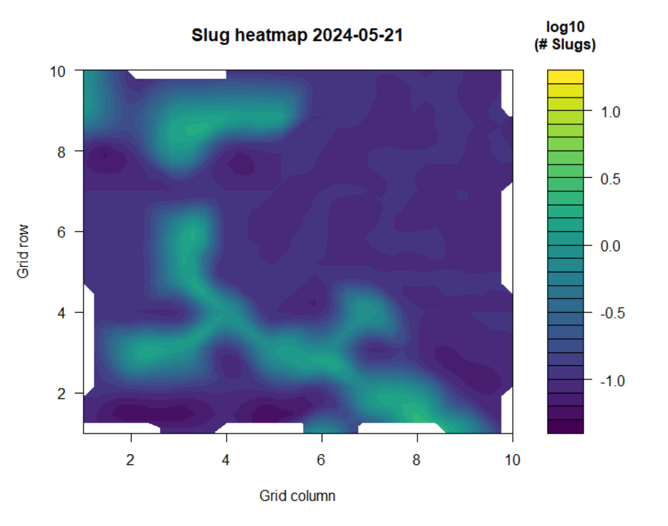

Ia = 1.324, Pa = 0.05 (random slug distribution)

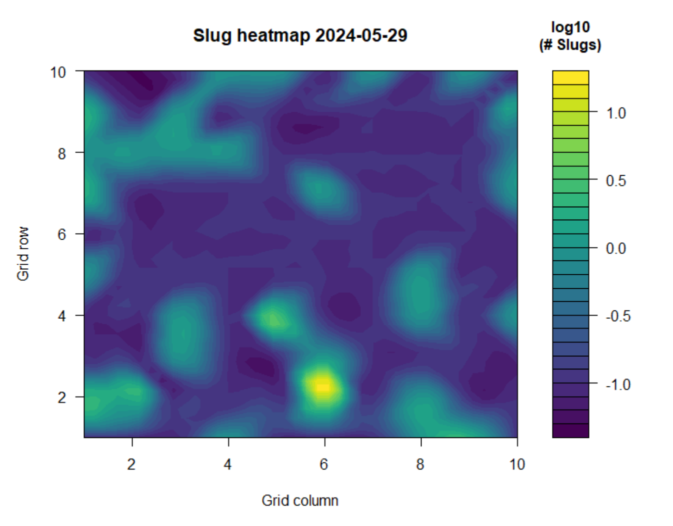

Ia = 0.933, Pa = 0.60 (random slug distribution)

Figure S3. The distribution of *Deroceras reticulatum* within the trapping grid at the Cambridgeshire 1 field site for each assessment date (slug numbers log-transformed). Ia = SADIE (Spatial Analysis by Distances Indices) index of aggregation; Pa = probability level. Slug distribution based on values of Ia and Pa is indicated in brackets.

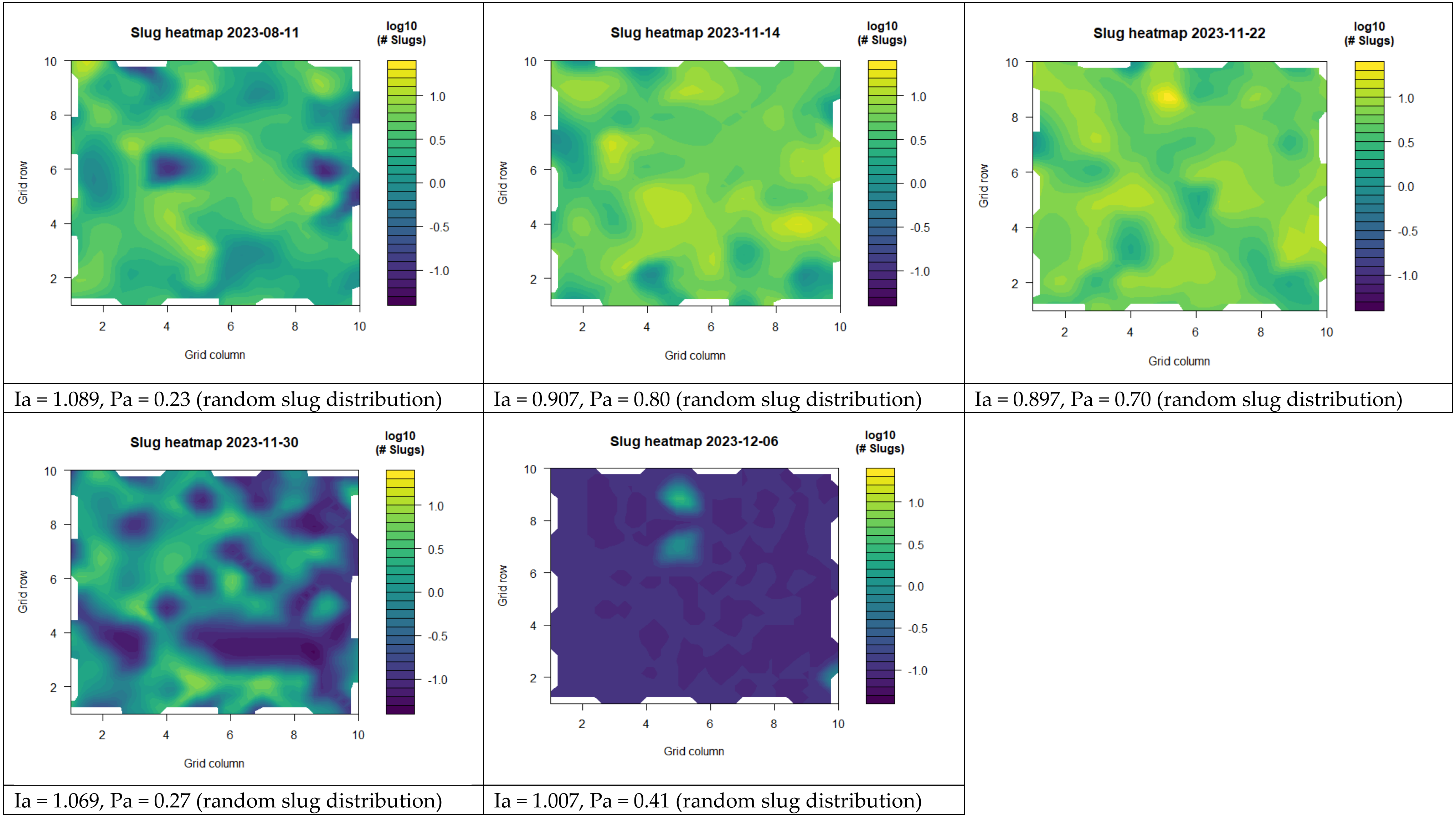

Figure S4. The distribution of *Deroceras reticulatum* within the trapping grid at the Cambridgeshire 2 field site for each assessment date (slug numbers log-transformed). Ia = SADIE (Spatial Analysis by Distances Indices) index of aggregation; Pa = probability level. Slug distribution based on values of Ia and Pa is indicated in brackets.

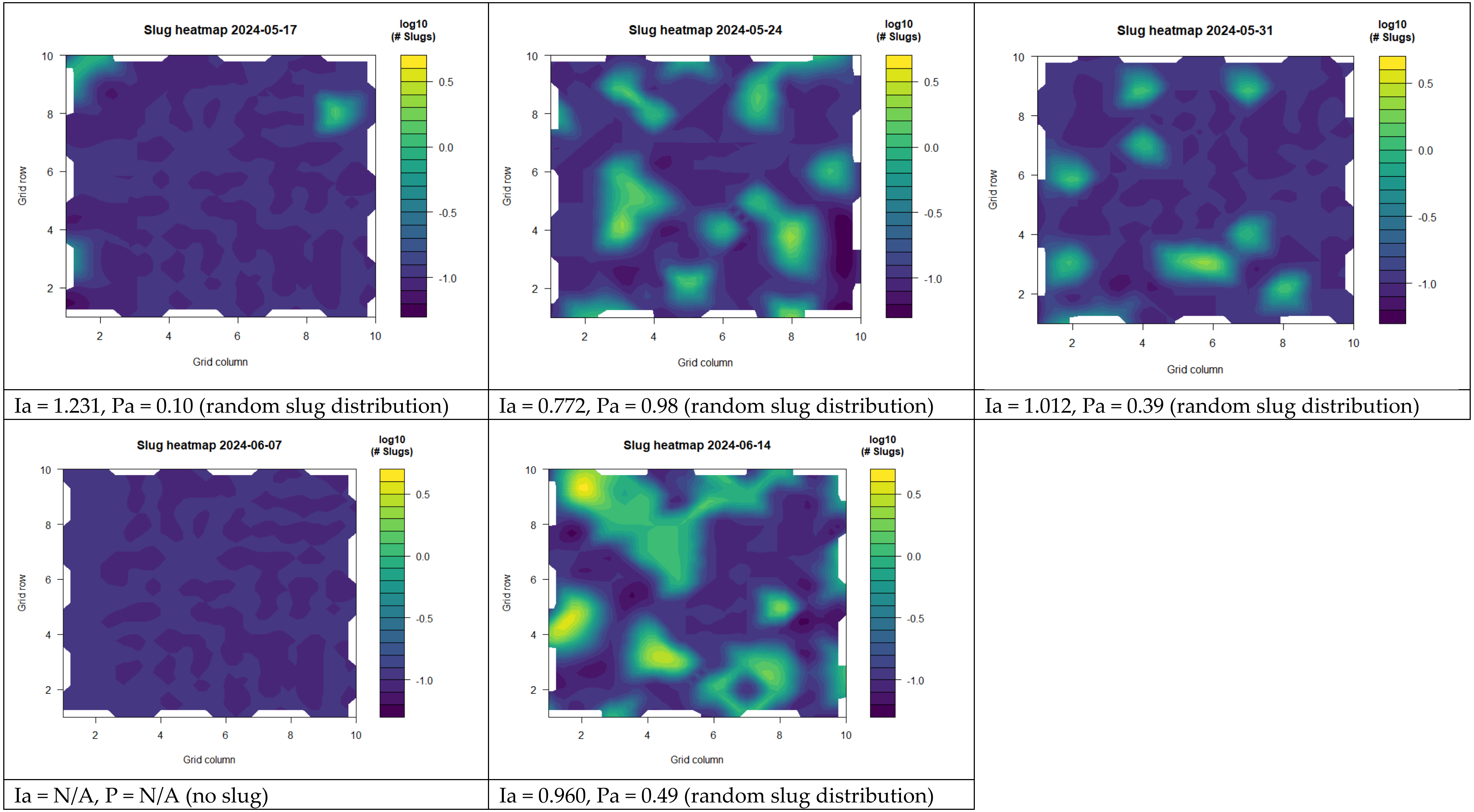

Figure S5. The distribution of *Deroceras reticulatum* within the trapping grid at the Hampshire field site for each assessment date (slug numbers log-transformed). Ia = SADIE (Spatial Analysis by Distances Indices) index of aggregation; Pa = probability level. Slug distribution based on values of Ia and Pa is indicated in brackets.

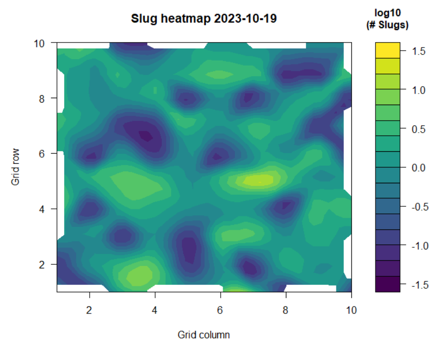

Ia = 0.816, Pa = 0.97 (random slug distribution)

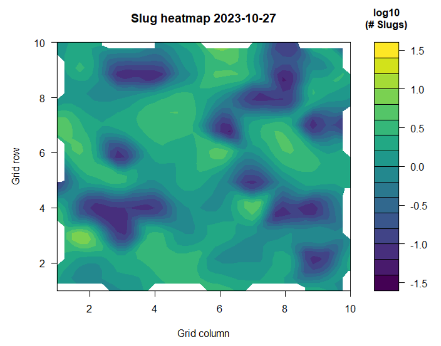

Ia = 0.941, Pa = 0.67 (random slug distribution)

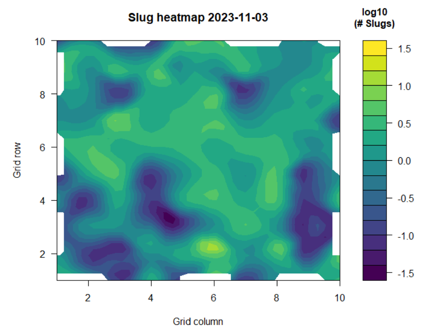

Ia = 1.089, Pa = 0.24 (random slug distribution)

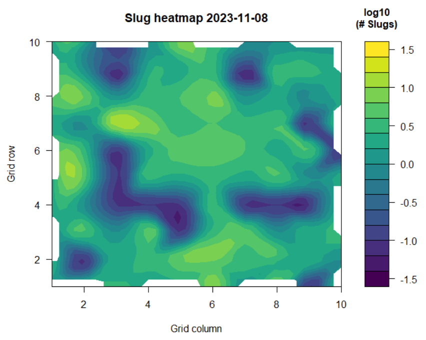

Ia = 0.955, Pa = 0.53 (random slug distribution)

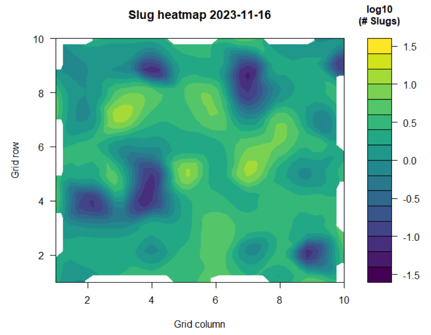

Ia = 1.129, Pa = 0.19 (random slug distribution)

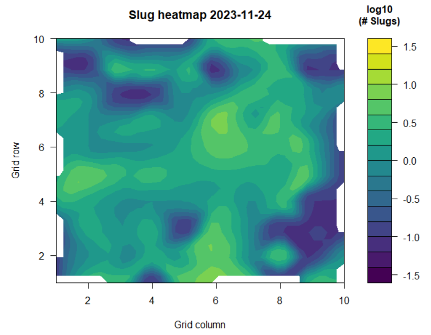

Ia = 1.210, Pa = 0.10 (random slug distribution)

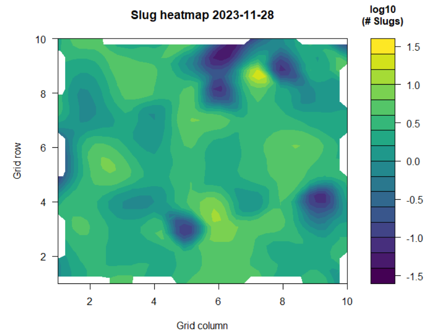

Ia = 0.100, Pa = 0.48 (random slug distribution)

Figure S6. The distribution of *Deroceras reticulatum* within the trapping grid at the Herefordshire field site for each assessment date (slug numbers log-transformed). Ia = SADIE (Spatial Analysis by Distances Indices) index of aggregation; Pa = probability level. Slug distribution based on values of Ia and Pa is indicated in brackets.

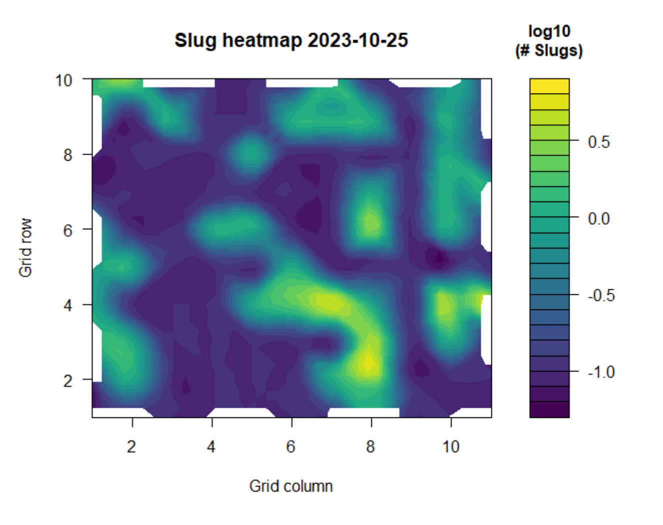

Ia = 1.069, Pa = 0.26 (random slug distribution)

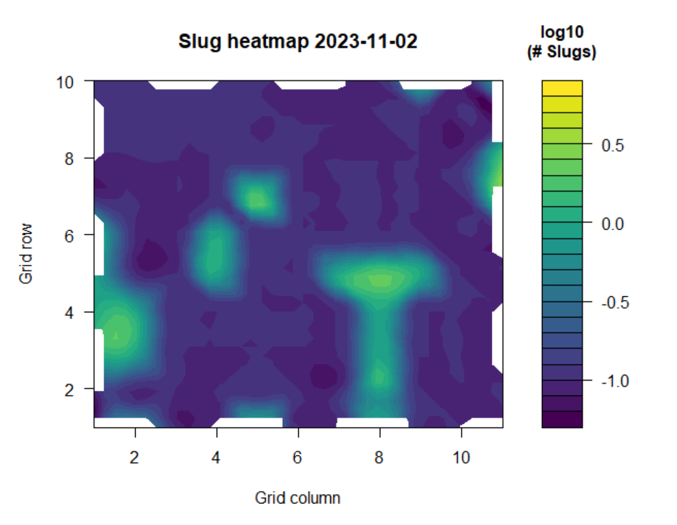

Ia = 1.079, Pa = 0.26 (random slug distribution)

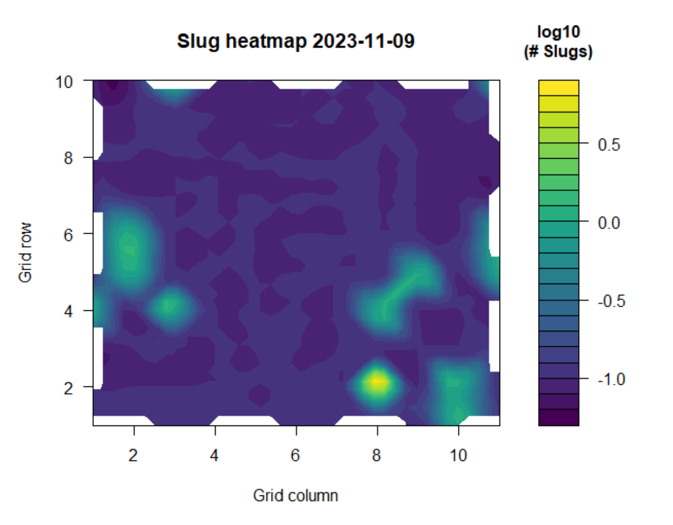

Ia = 1.044, Pa = 0.36 (random slug distribution)

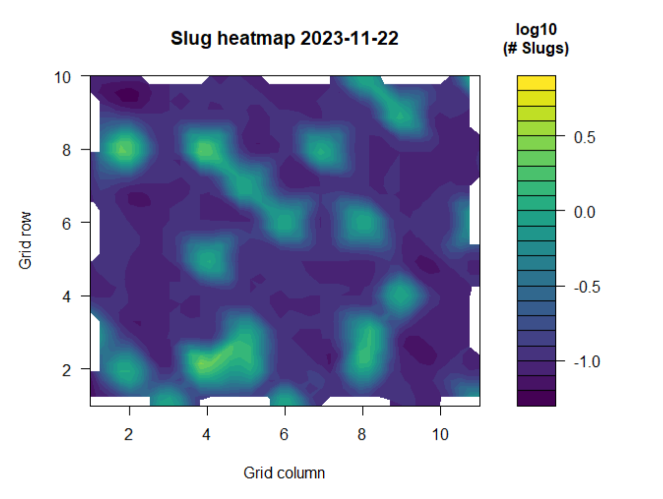

Ia = 0.857, Pa = 0.83 (random slug distribution)

Figure S7. The distribution of *Deroceras reticulatum* within the trapping grid at the Hertfordshire 1 field site for each assessment date (slug numbers log-transformed). Ia = SADIE (Spatial Analysis by Distances Indices) index of aggregation; Pa = probability level. Slug distribution based on values of Ia and Pa is indicated in brackets.

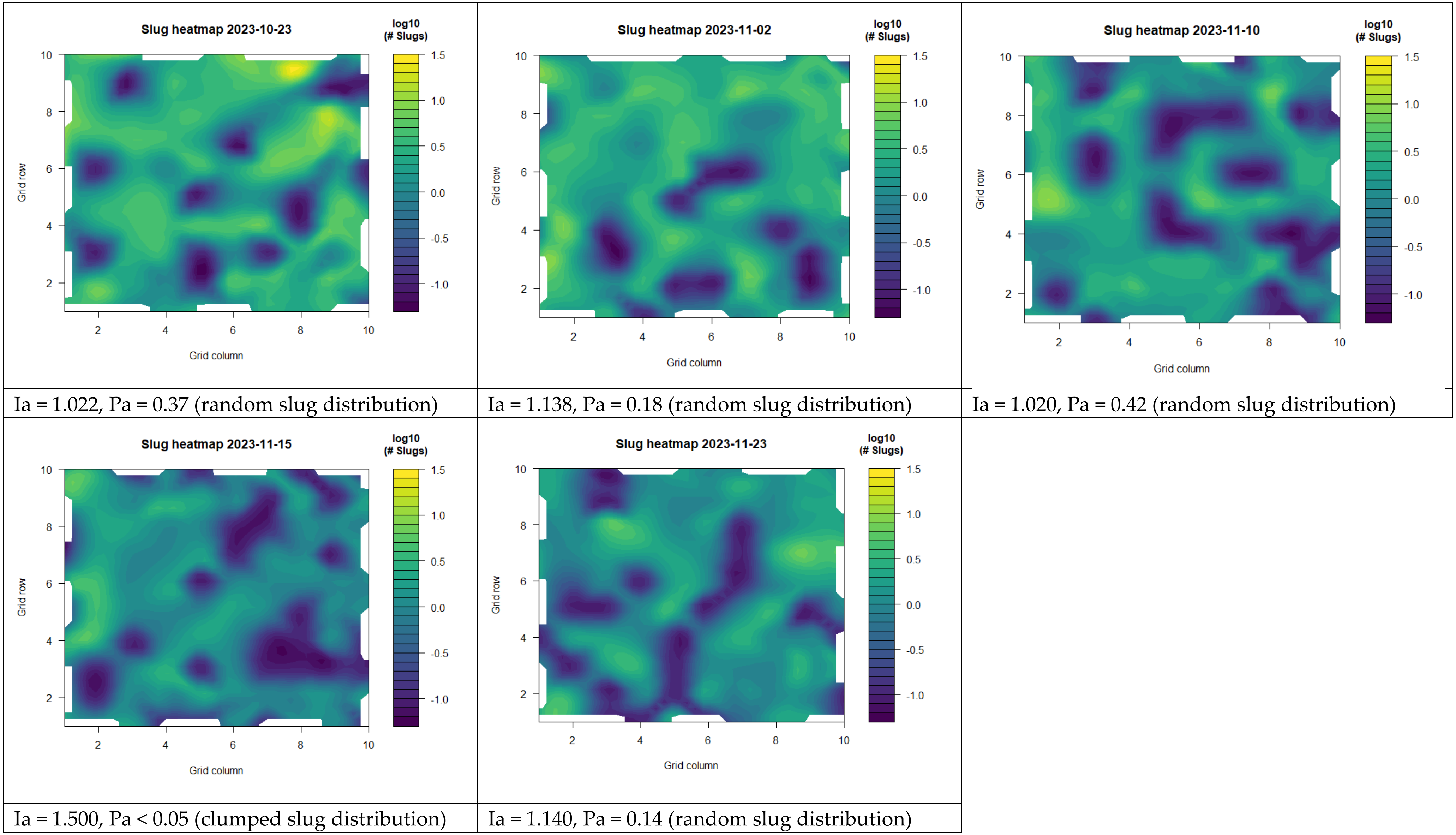

Ia = 1.022, Pa = 0.37 (random slug distribution)

Ia = 1.138, Pa = 0.18 (random slug distribution)

Ia = 1.020, Pa = 0.42 (random slug distribution)

Ia = 1.500, Pa < 0.05 (clumped slug distribution)

Ia = 1.140, Pa = 0.14 (random slug distribution)

Figure S8. The distribution of *Deroceras reticulatum* within the trapping grid at the Hertfordshire 2 field site for each assessment date (slug numbers log-transformed). Ia = SADIE (Spatial Analysis by Distances Indices) index of aggregation; Pa = probability level. Slug distribution based on values of Ia and Pa is indicated in brackets.

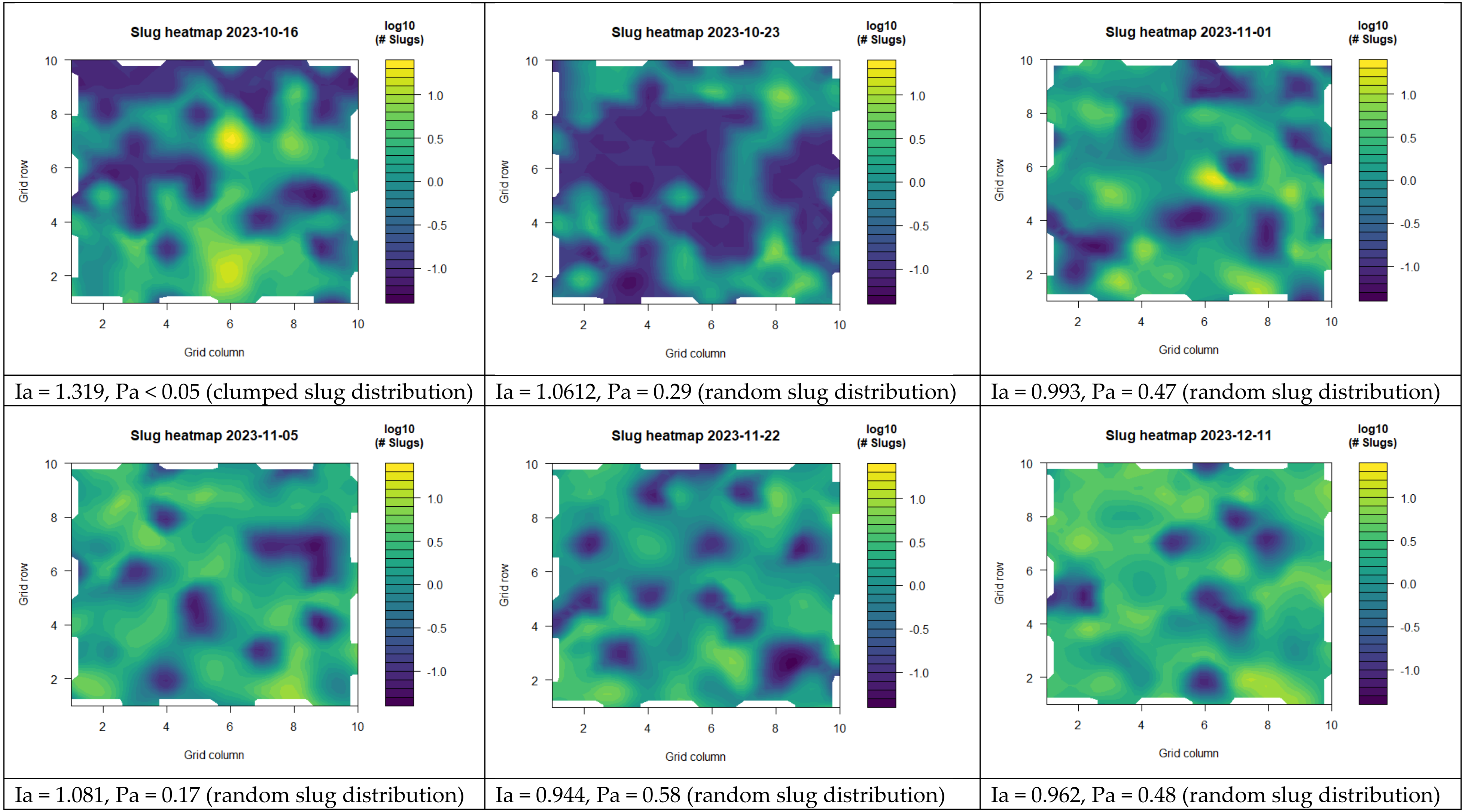

Figure S9. The distribution of *Deroceras reticulatum* within the trapping grid at the Kent field site for each assessment date (slug numbers log-transformed). Ia = SADIE (Spatial Analysis by Distances Indices) index of aggregation; Pa = probability level. Slug distribution based on values of Ia and Pa is indicated in brackets.

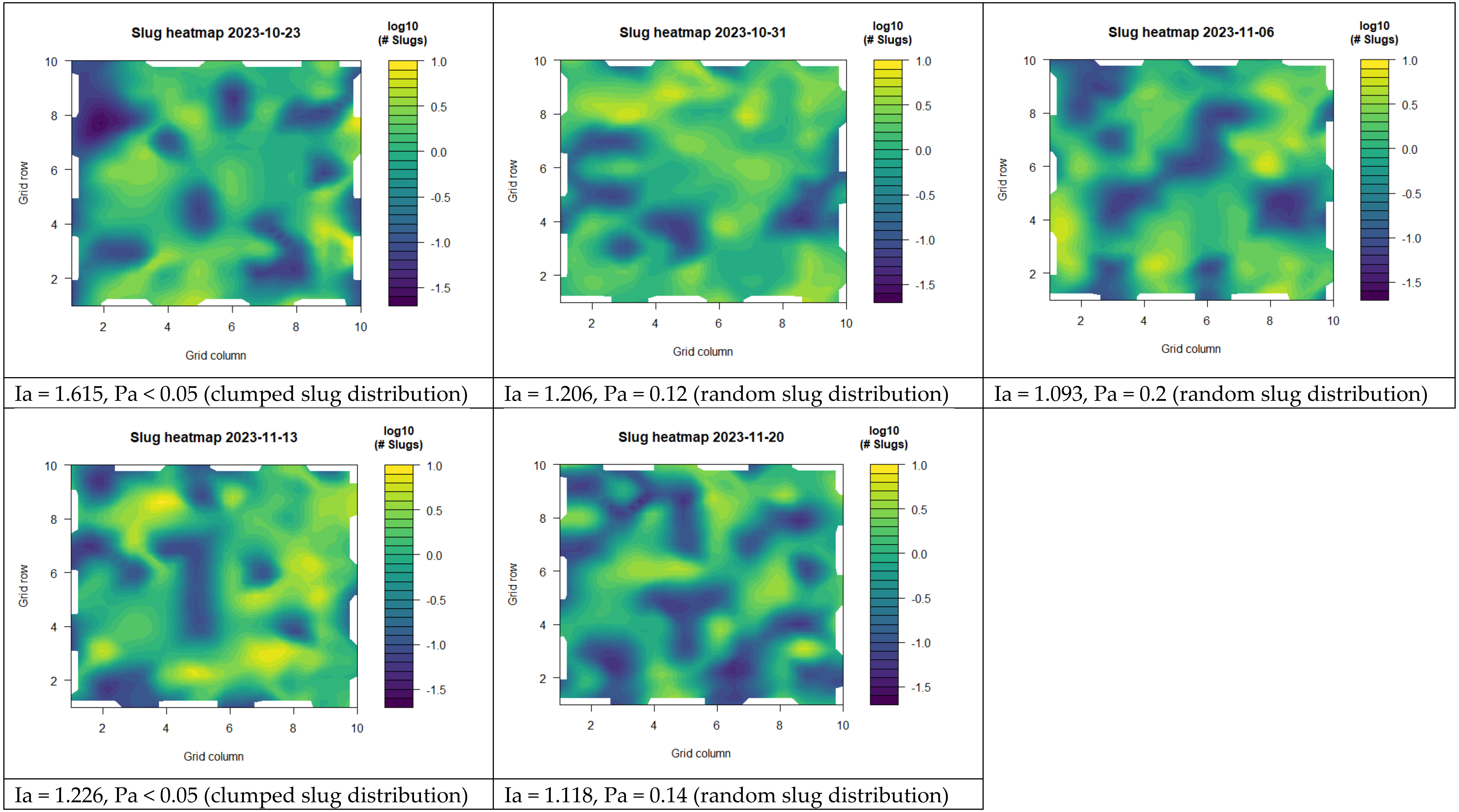

Figure S10. The distribution of *Deroceras reticulatum* within the trapping grid at the Leicestershire field site for each assessment date (slug numbers log-transformed). Ia = SADIE (Spatial Analysis by Distances Indices) index of aggregation; Pa = probability level. Slug distribution based on values of Ia and Pa is indicated in brackets.

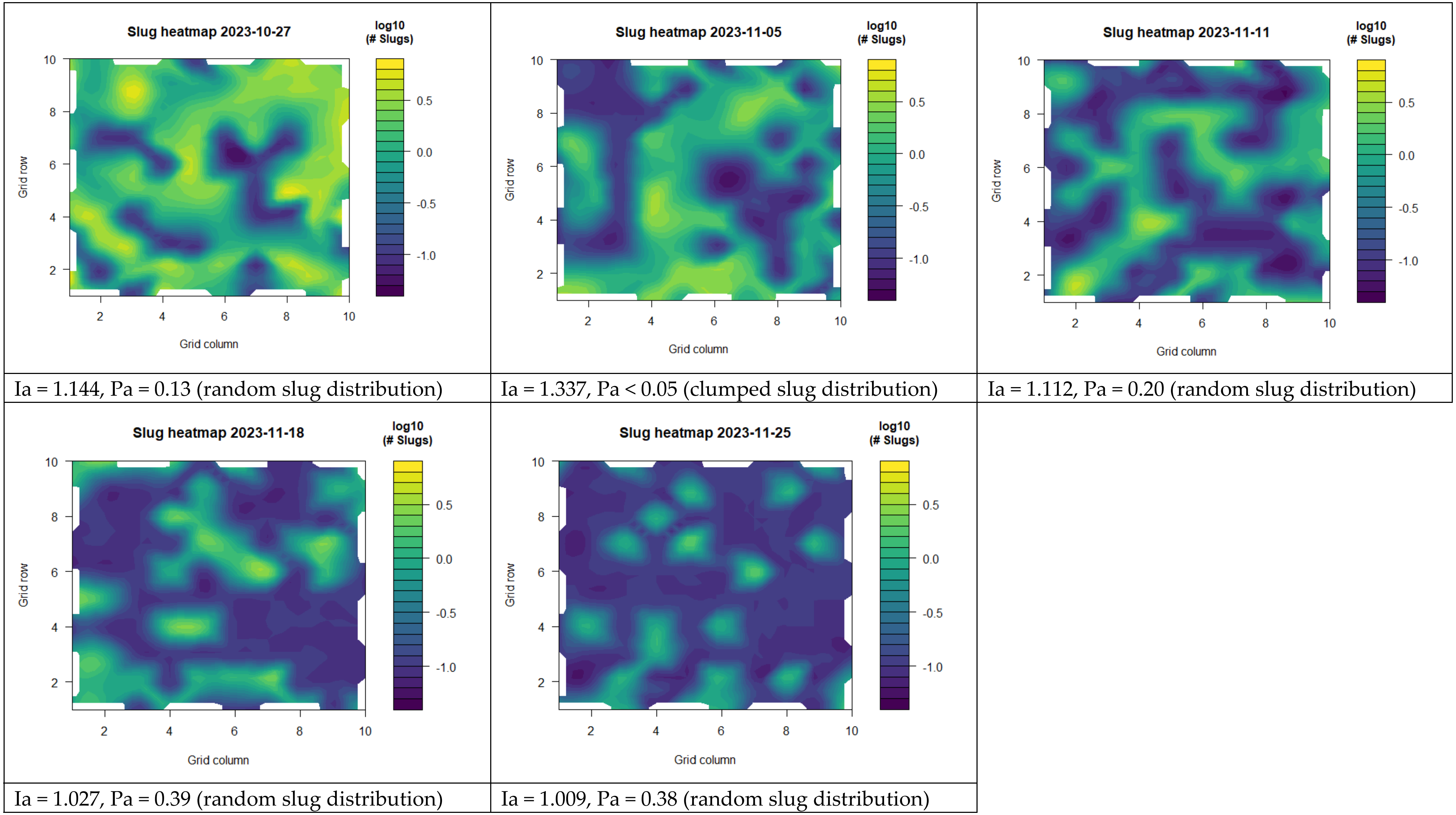

Figure S11. The distribution of *Deroceras reticulatum* within the trapping grid at the Lincolnshire 1 field site for each assessment date (slug numbers log-transformed). Ia = SADIE (Spatial Analysis by Distances Indices) index of aggregation; Pa = probability level. Slug distribution based on values of Ia and Pa is indicated in brackets.

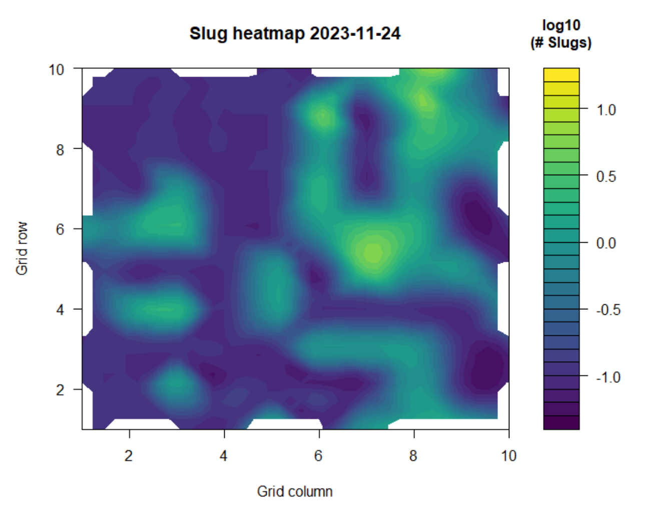

Ia = 1.259, Pa = 0.07 (random slug distribution)

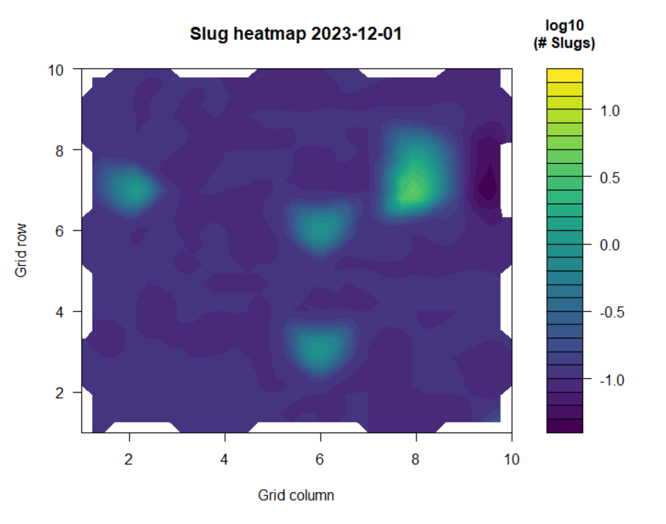

Ia = 0.991, Pa = 0.44 (random slug distribution)

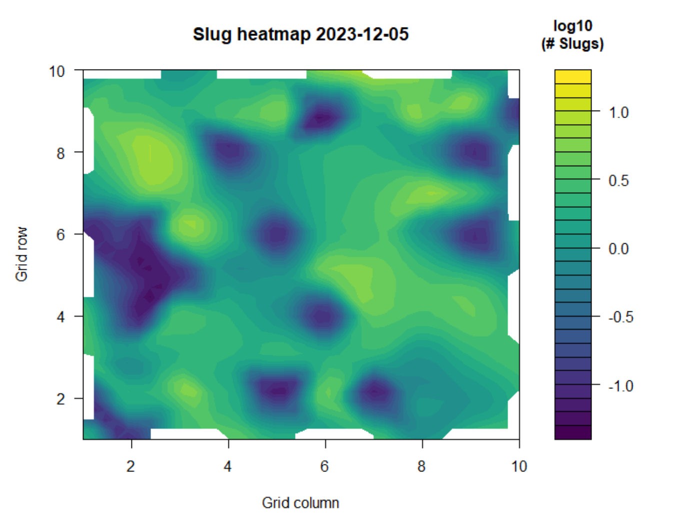

Ia = 1.179, Pa = 0.09 (random slug distribution)

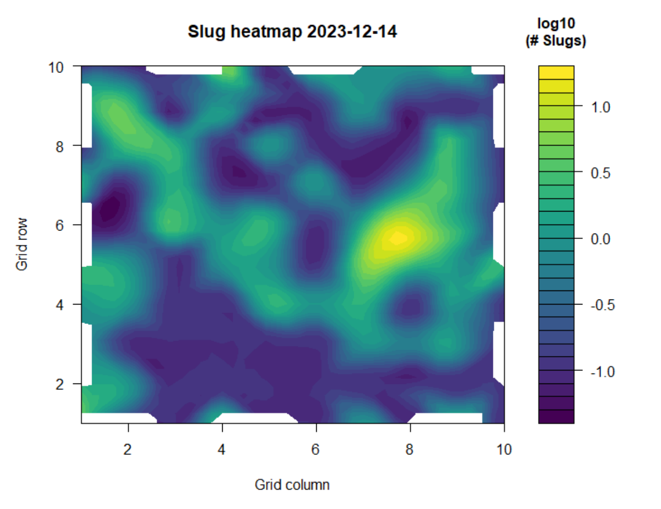

Ia = 0.956, Pa = 0.53 (random slug distribution)

Figure S12. The distribution of *Deroceras reticulatum* within the trapping grid at the Lincolnshire 2 field site for each assessment date (slug numbers log-transformed). Ia = SADIE (Spatial Analysis by Distances Indices) index of aggregation; Pa = probability level. Slug distribution based on values of Ia and Pa is indicated in brackets.

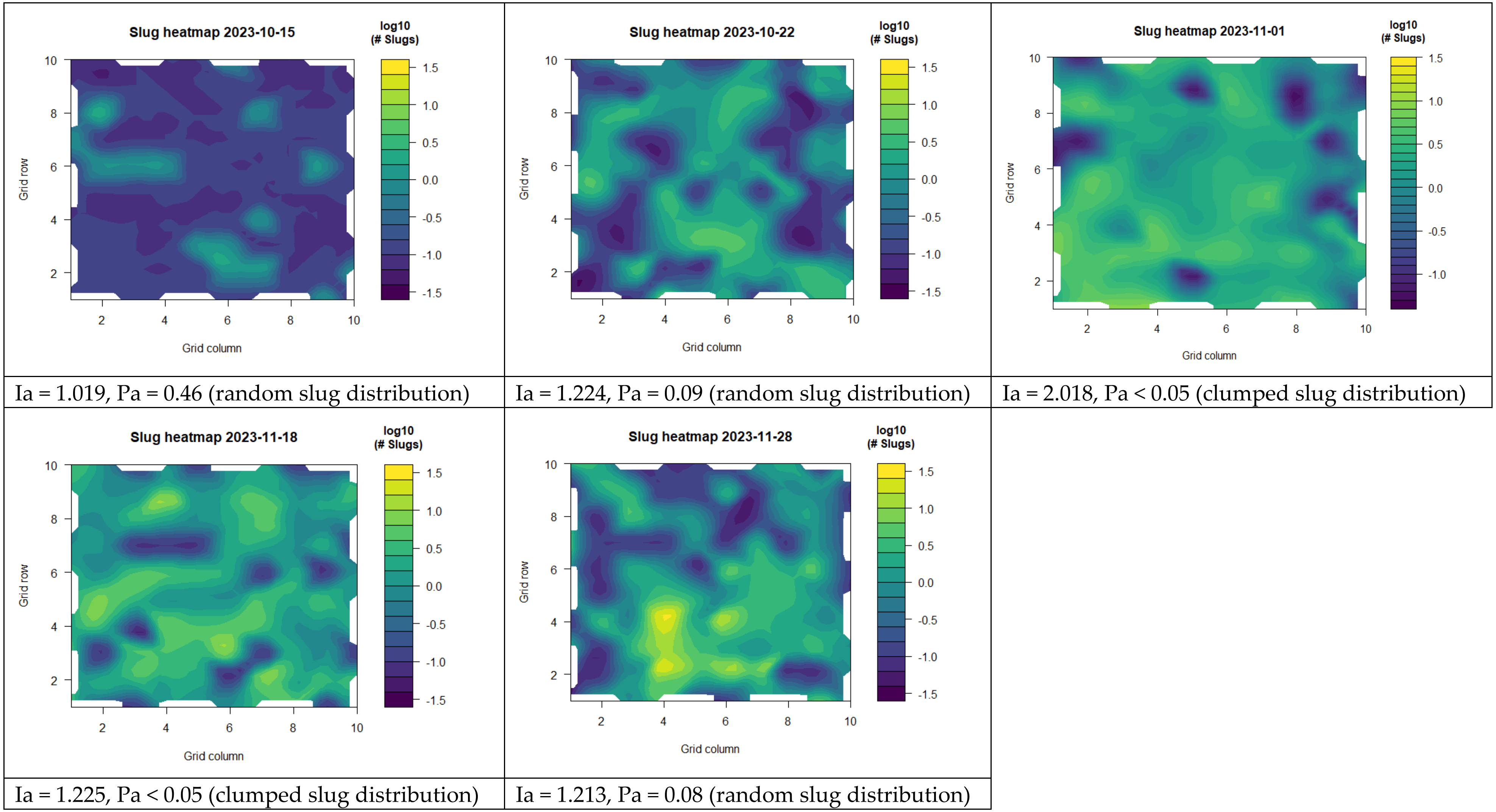

Figure S13. The distribution of *Deroceras reticulatum* within the trapping grid at the North Yorkshire field site for each assessment date (slug numbers log-transformed). Ia = SADIE (Spatial Analysis by Distances Indices) index of aggregation; Pa = probability level. Slug distribution based on values of Ia and Pa is indicated in brackets.

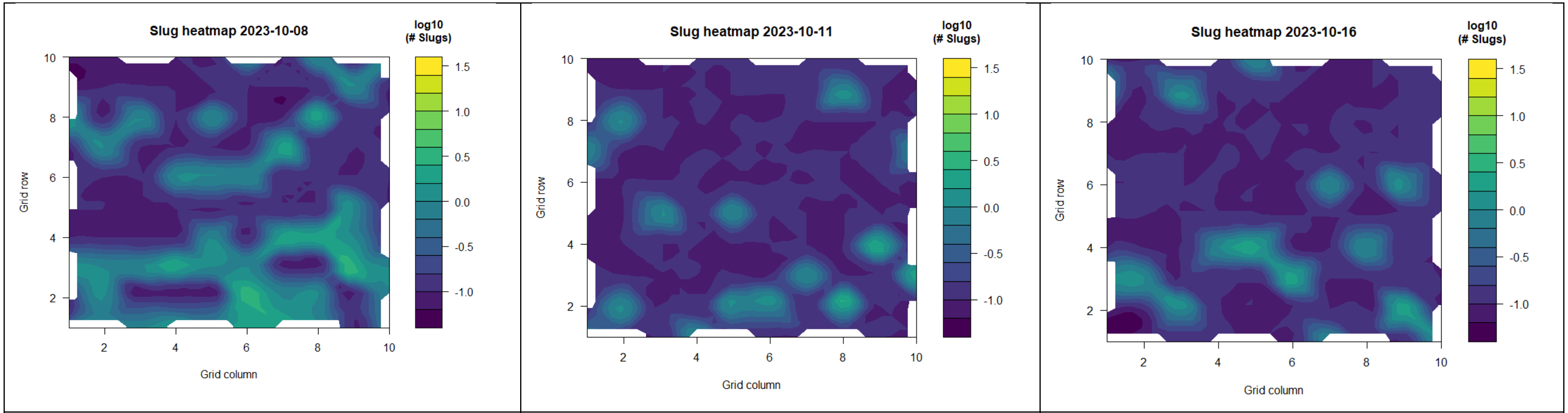

Ia = 1.525, Pa < 0.05 (clumped slug distribution)	Ia = 1.180, Pa = 0.12 (random slug distribution)	Ia = 1.058, Pa = 0.28 (random slug distribution)

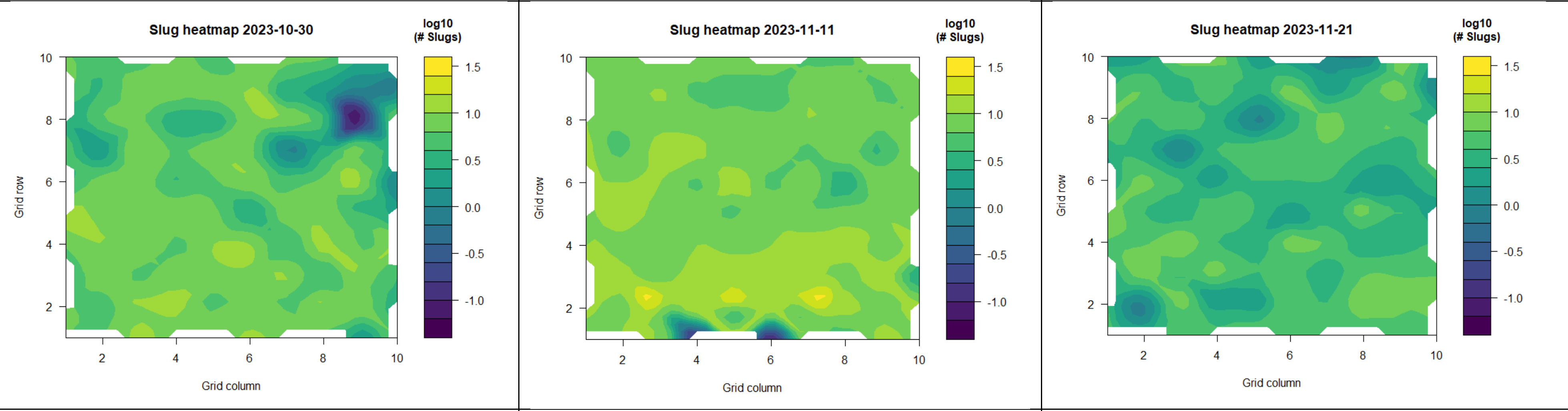

Ia = 1.613, Pa < 0.05 (clumped slug distribution)	Ia = 1.381, Pa < 0.05 (clumped slug distribution)	Ia = 1.182, Pa = 0.15 (random slug distribution)

Figure S14. The distribution of *Deroceras reticulatum* within the trapping grid at the Northamptonshire field site for each assessment date (slug numbers log-transformed). Ia = SADIE (Spatial Analysis by Distances Indices) index of aggregation; Pa = probability level. Slug distribution based on values of Ia and Pa is indicated in brackets.

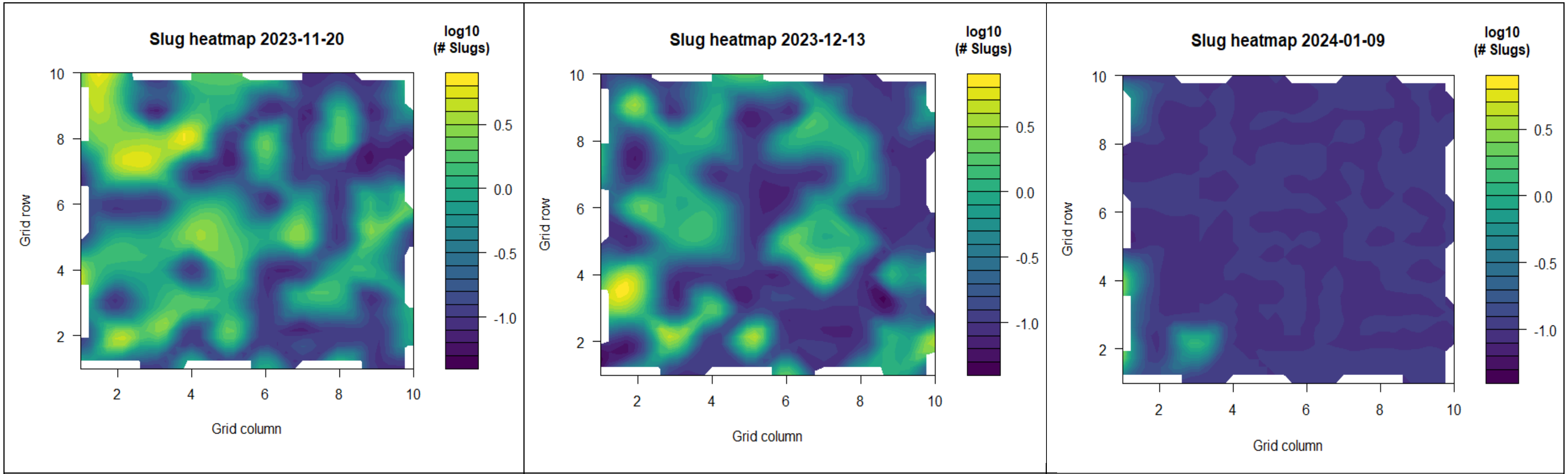

Ia = 1.387, P < 0.05 (clumped slug distribution)	Ia = 1.099, Pa = 0.19 (random slug distribution)	Ia = 1.741, Pa = 0.01 (clumped slug distribution)

Figure S15. The distribution of *Deroceras reticulatum* within the trapping grid at the Nottinghamshire field site for each assessment date (slug numbers log-transformed). Ia = SADIE (Spatial Analysis by Distances Indices) index of aggregation; Pa = probability level. Slug distribution based on values of Ia and Pa is indicated in brackets.

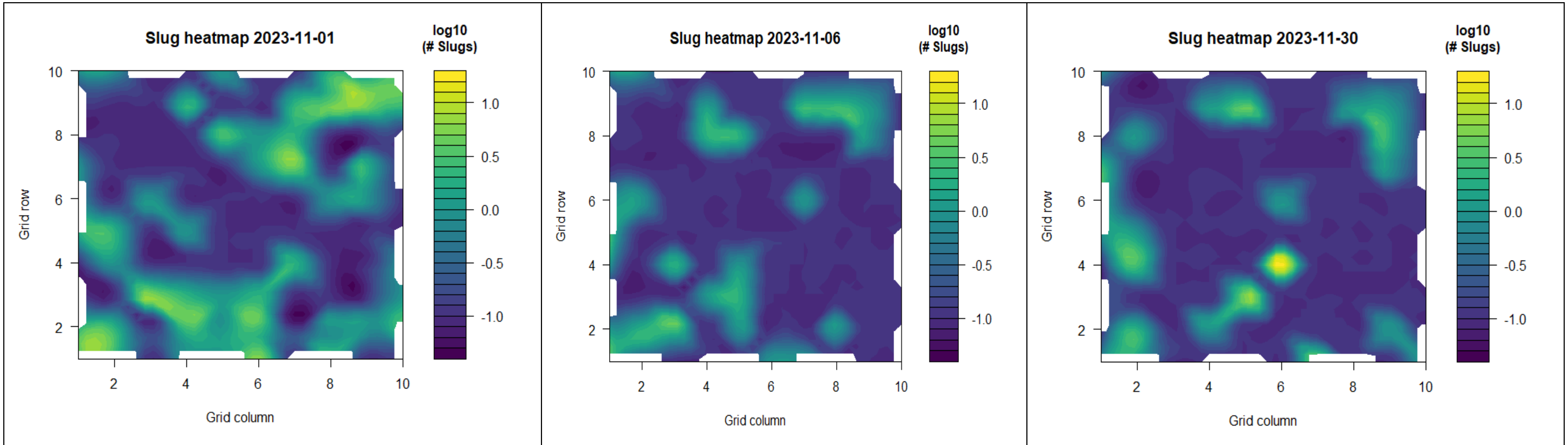

Ia = 1.095, Pa = 0.17 (random slug distribution)	Ia = 1.402, Pa < 0.05 (clumped slug distribution)	Ia = 0.758, Pa = 0.97 (random slug distribution)

Figure S16. The distribution of *Deroceras reticulatum* within the trapping grid at the Oxfordshire 1 field site for each assessment date (slug numbers log-transformed). Ia = SADIE (Spatial Analysis by Distances Indices) index of aggregation; Pa = probability level. Slug distribution based on values of Ia and Pa is indicated in brackets.

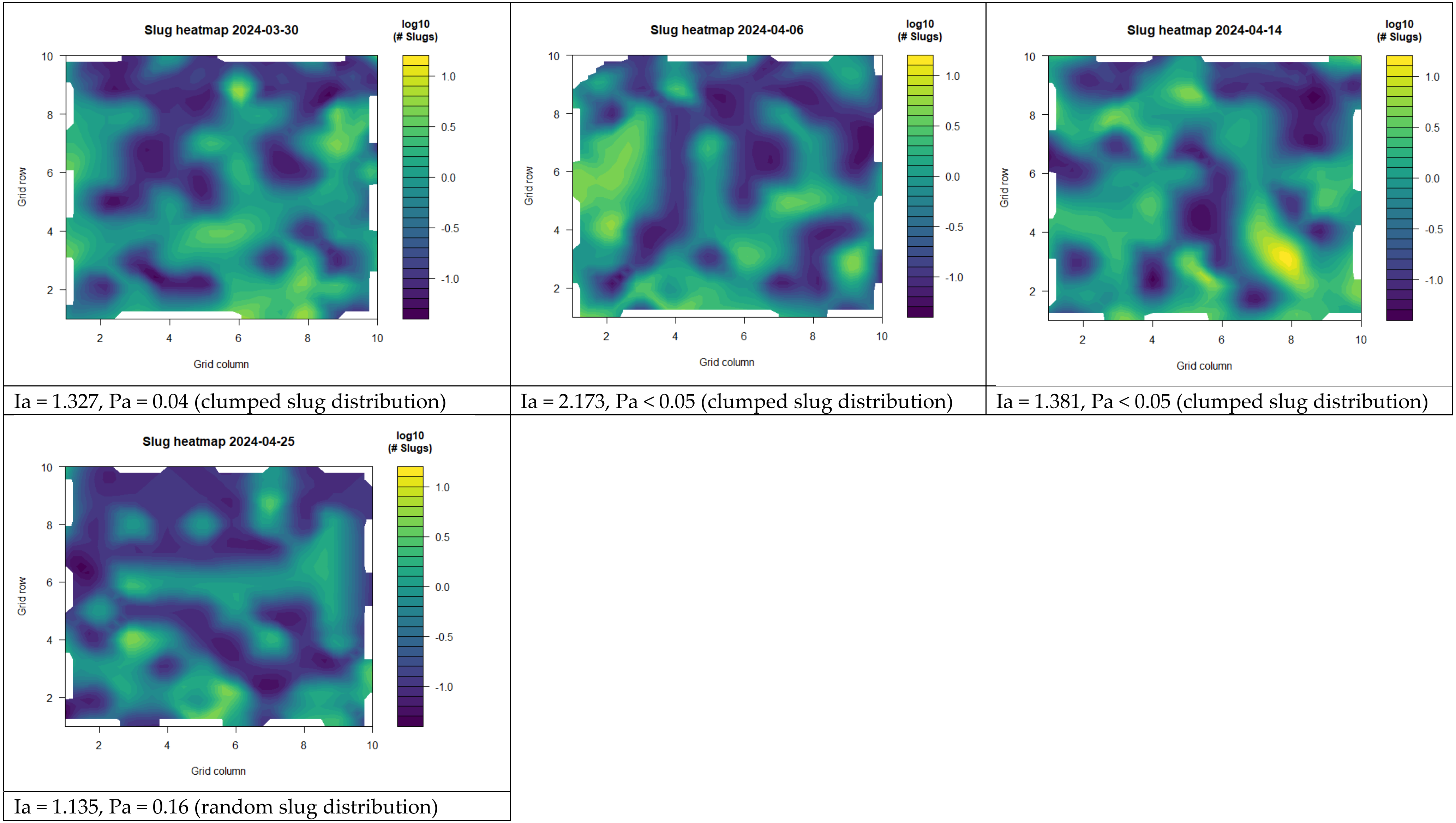

Figure S17. The distribution of *Deroceras reticulatum* within the trapping grid at the Oxfordshire 2 field site for each assessment date (slug numbers log-transformed). Ia = SADIE (Spatial Analysis by Distances Indices) index of aggregation; Pa = probability level. Slug distribution based on values of Ia and Pa is indicated in brackets.

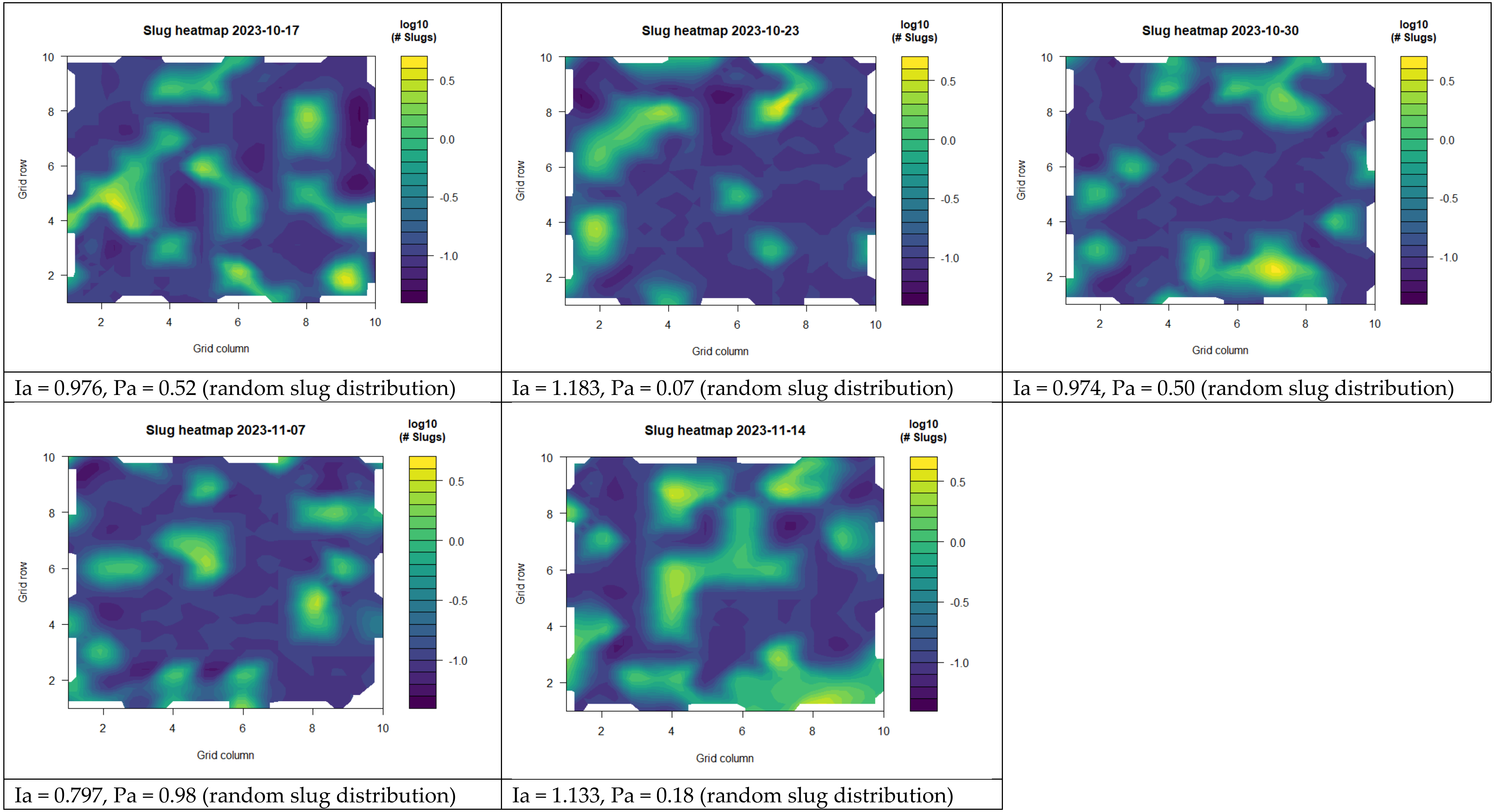

Figure S18. The distribution of *Deroceras reticulatum* within the trapping grid at the Rutland field site for each assessment date (slug numbers log-transformed). Ia = SADIE (Spatial Analysis by Distances Indices) index of aggregation; Pa = probability level. Slug distribution based on values of Ia and Pa is indicated in brackets.

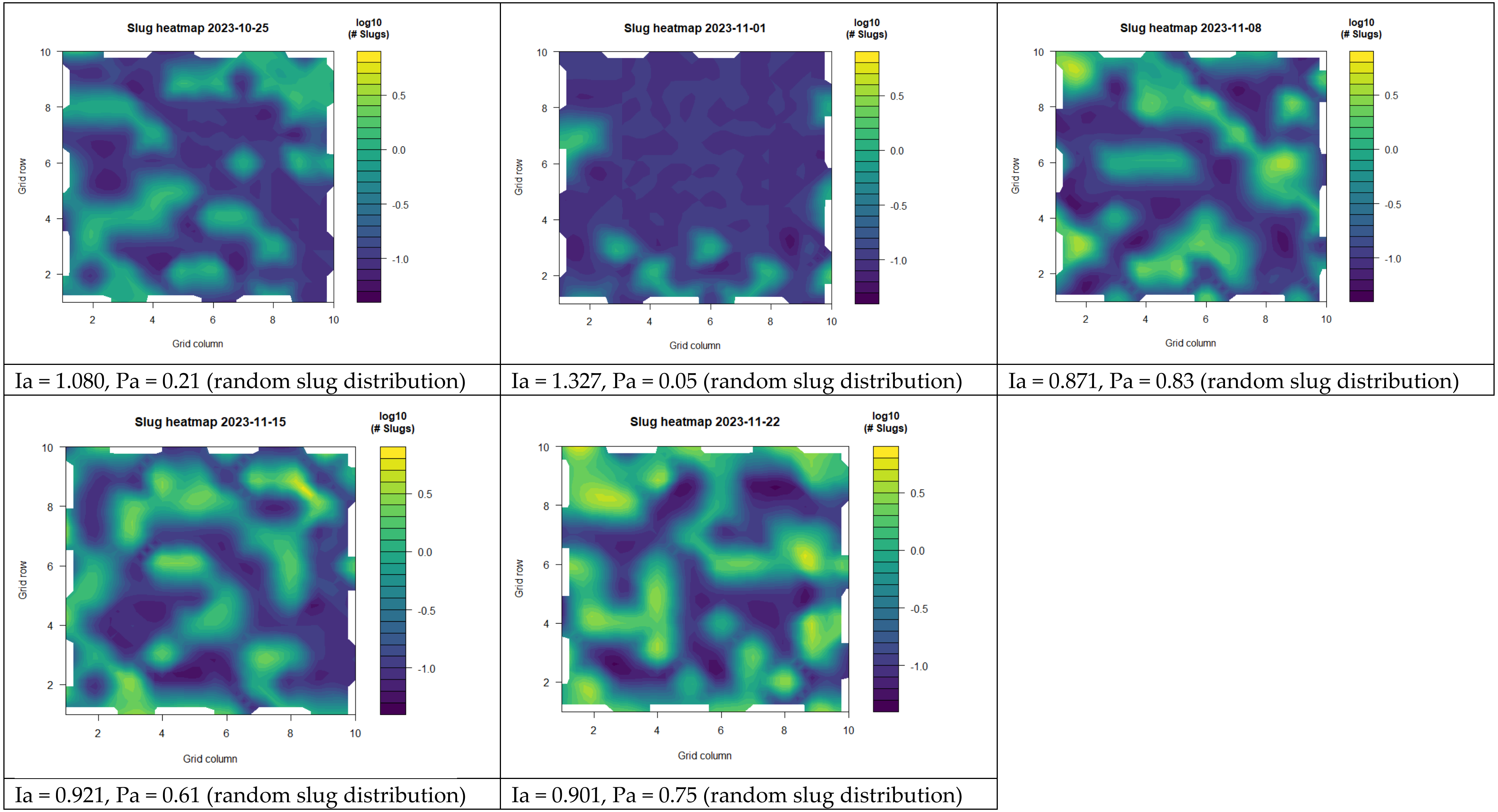

Figure S19. The distribution of *Deroceras reticulatum* within the trapping grid at the Shropshire field site for each assessment date (slug numbers log-transformed). Ia = SADIE (Spatial Analysis by Distances Indices) index of aggregation; Pa = probability level. Slug distribution based on values of Ia and Pa is indicated in brackets.

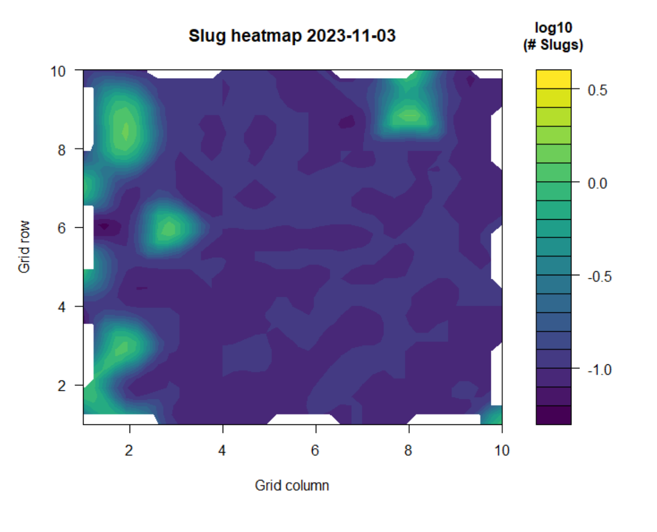

Ia = 1.281, Pa < 0.05 (clumped slug distribution)

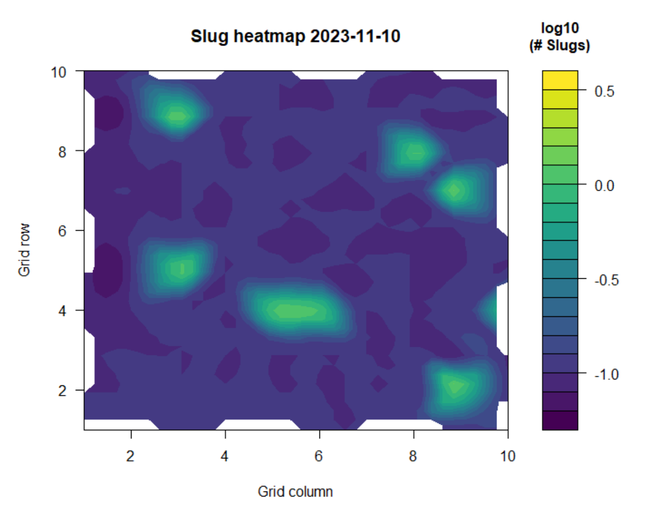

Ia = 0.863, Pa = 0.85 (random slug distribution)

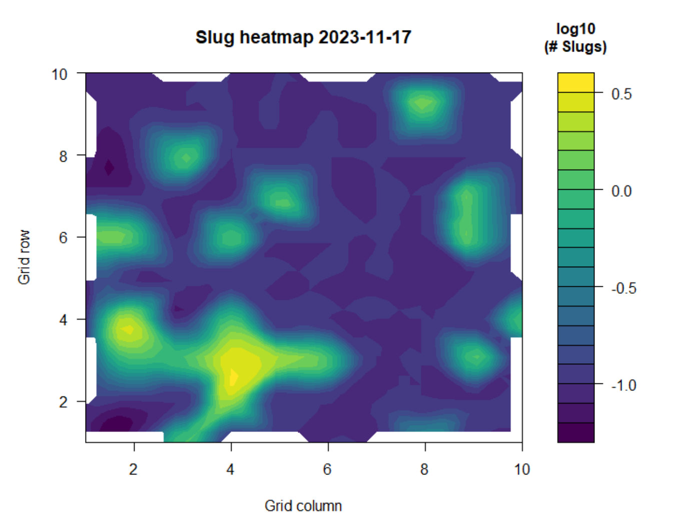

Ia = 1.191, Pa = 0.12 (random slug distribution)

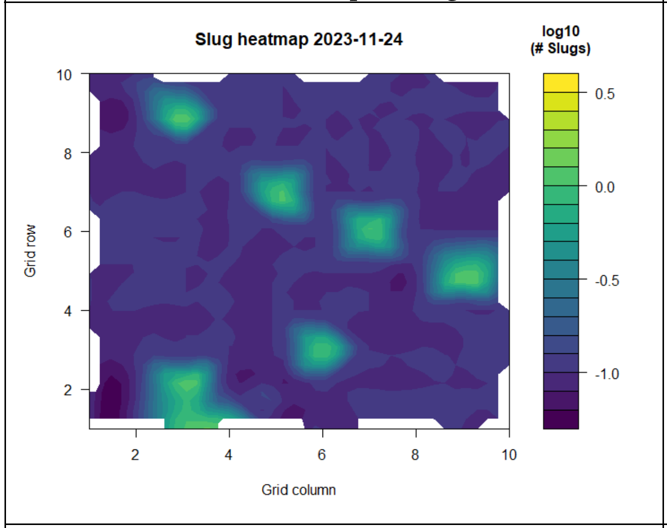

Ia = 1.012, Pa = 0.38 (random slug distribution)

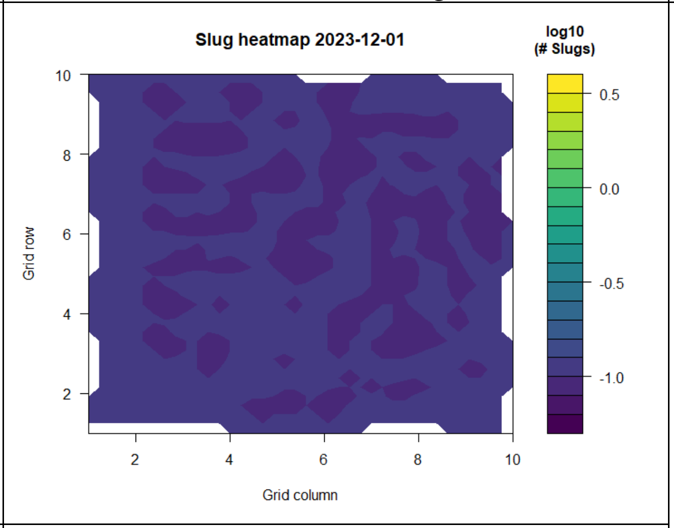

Ia = N/A, P = N/A (no slug)

Figure S20. The distribution of *Deroceras reticulatum* within the trapping grid at the Suffolk field site for each assessment date (slug numbers log-transformed). Ia = SADIE (Spatial Analysis by Distances Indices) index of aggregation; Pa = probability level. Slug distribution based on values of Ia and Pa is indicated in brackets.

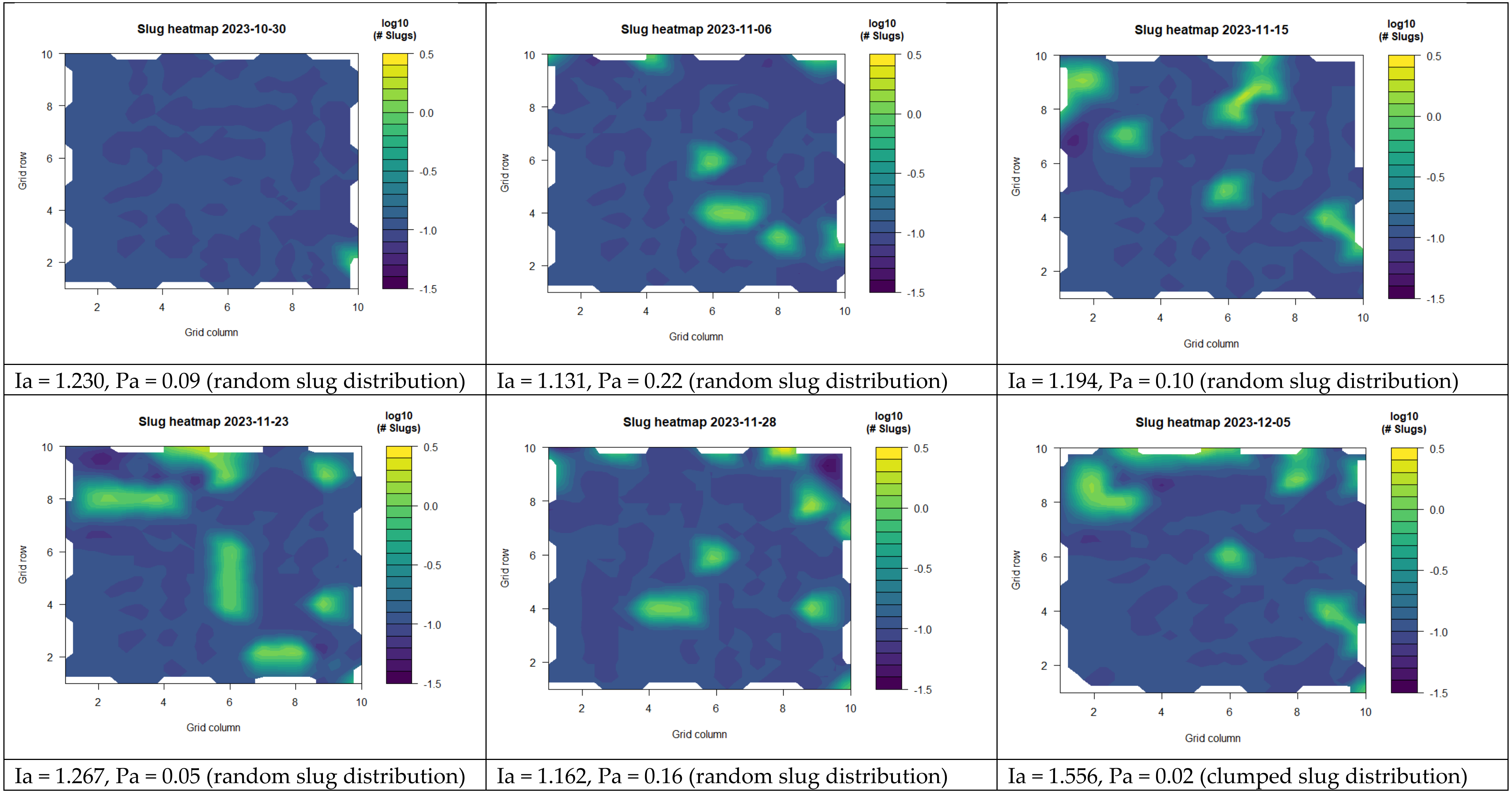

Figure S21. The distribution of *Deroceras reticulatum* within the trapping grid at the West Sussex field site for each assessment date (slug numbers log-transformed). Ia = SADIE (Spatial Analysis by Distances Indices) index of aggregation; Pa = probability level. Slug distribution based on values of Ia and Pa is indicated in brackets.

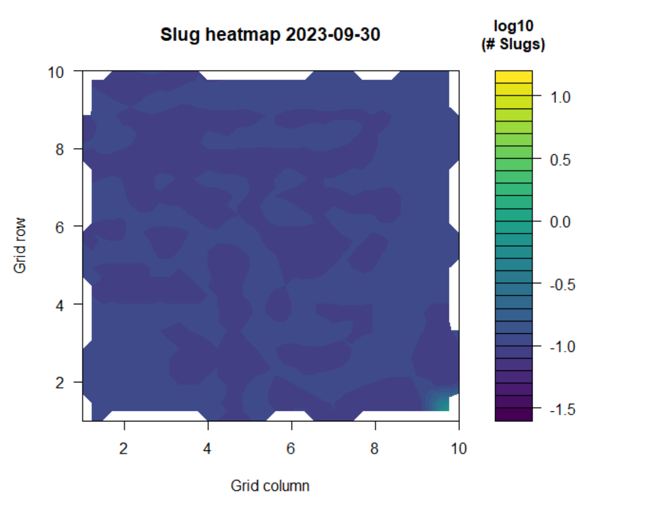	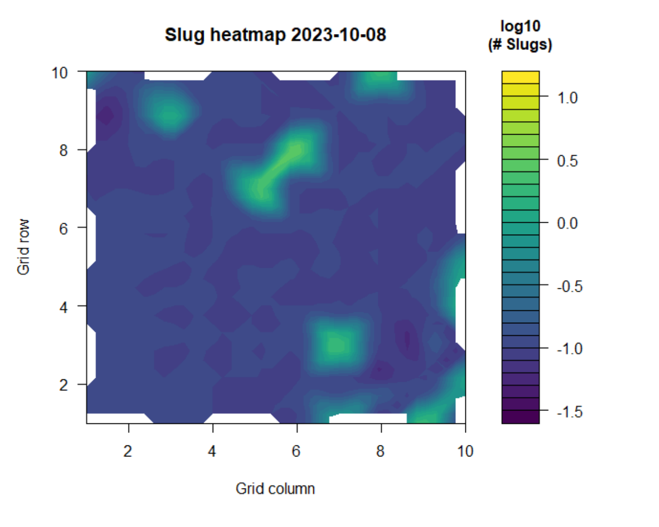	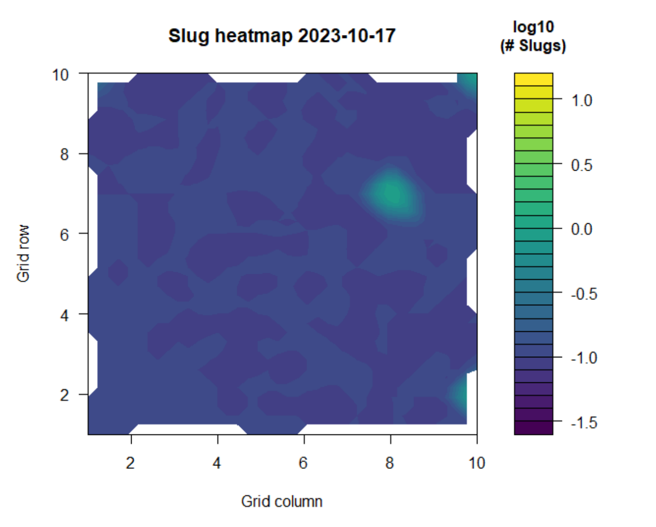
Ia = 1.340, Pa < 0.05 (clumped slug distribution)	Ia = 1.163, Pa = 0.08 (random slug distribution)	Ia = 1.137, Pa = 0.23 (random slug distribution)
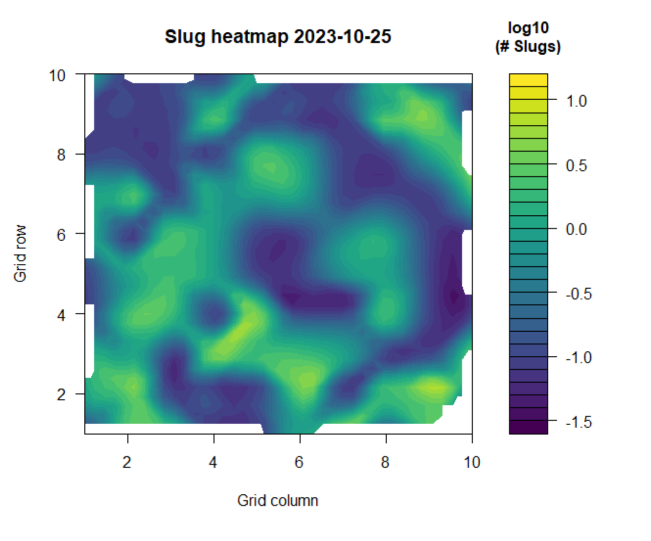	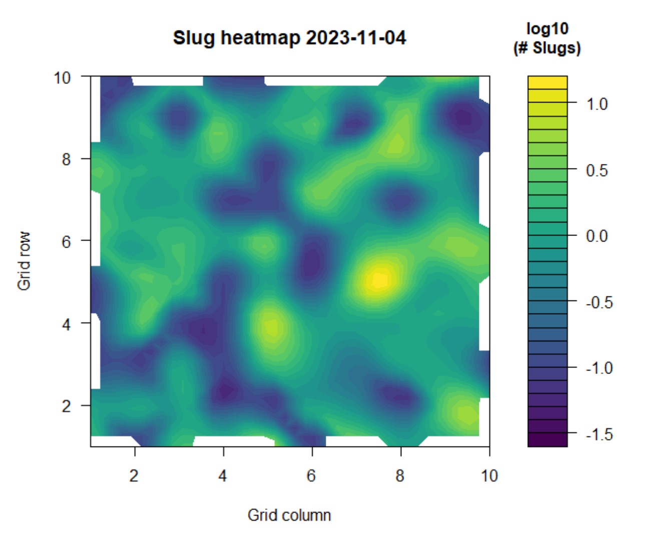	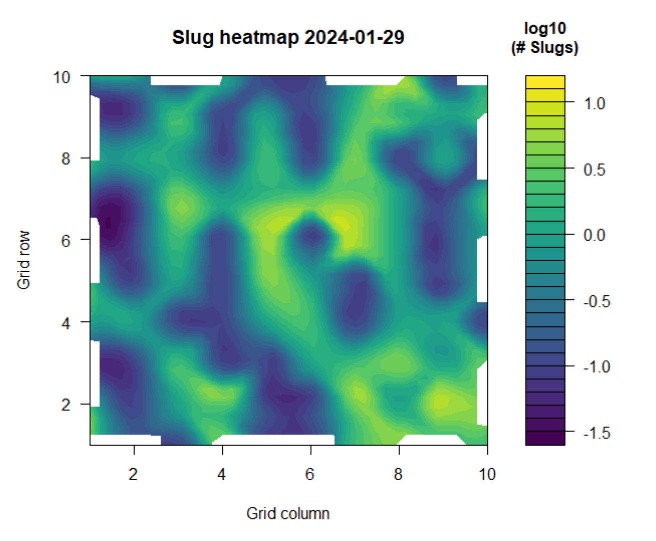
Ia = 1.198, Pa = 0.05 (random slug distribution)	Ia = 1.049, Pa = 0.3 (random slug distribution)	Ia = 1.342, Pa = 0.03 (clumped slug distribution)

Figure S22. The distribution of *Deroceras reticulatum* within the trapping grid at the Yorkshire field site for each assessment date (slug numbers log-transformed). Ia = SADIE (Spatial Analysis by Distances Indices) index of aggregation; Pa = probability level. Slug distribution based on values of Ia and Pa is indicated in brackets.
